# A specific and adaptable approach to track CD206^+^ macrophages by molecular MRI and fluorescence imaging

**DOI:** 10.7150/thno.96488

**Published:** 2025-01-01

**Authors:** Cuihua Wang, Negin Jalali Motlagh, Gregory R. Wojtkiewicz, Hongzhi Yang, Hyung-Hwan Kim, John W. Chen

**Affiliations:** 1 Institute for Innovation in Imaging, Department of Radiology, Massachusetts General Hospital, Charlestown, Massachusetts, 02129, MA.; 2 Center for Systems Biology, Massachusetts General Hospital and Harvard Medical School, 185 Cambridge St, Boston, MA.; 3 Stroke and Neurovascular Regulation Laboratory, Massachusetts General Hospital, Boston, MA

**Keywords:** molecular magnetic resonance imaging, mannose receptor (CD206), tumor-associated macrophages, wound healing, glioma, stroke.

## Abstract

**Rationale:** The mannose receptor (CD206, expressed by the gene *Mrc1*) is a surface marker overexpressed by anti-inflammatory and pro-tumoral macrophages. As such, CD206^+^ macrophages play key roles in the immune response to different pathophysiological conditions and represent a promising diagnostic and therapeutic target. However, methods to specifically target these cells remain challenging. In this study, we describe a multi-mannose approach to develop CD206-targeting fluorescent and MRI agents that specifically and sensitively detect and monitor CD206^+^ macrophage immune response in different disease conditions.

**Methods:** We designed and synthesized fluorescent agents MR1-cy5 and MR2-cy5, and MRI agents Mann2-DTPA-Gd and MannGdFish. Cellular assays using pro-inflammatory and anti-inflammatory macrophages differentiated from RAW 264.7 cells were performed, and signals were detected by fluorescence microscopy and inductively coupled plasma mass spectrometry (ICP-MS) to validate specificity *in vitro*. *In vivo* specificity and efficacy of the agents were evaluated by MRI in a subcutaneous wound healing model and experimental glioma with *Mrc1*^+/+^ without and with D-mannose treatment, *Mrc1*^+/-^, and *Mrc1*^-/-^ mice, and in stroke. One-way ANOVA and two-way ANOVA tests were used for data analysis. P < 0.05 was considered statistically different.

**Results:** Both *in vitro* fluorescence imaging with MR2-cy5, ICP-MS with Mann2-DTPA-Gd, and *in vivo* MRI in *Mrc1*^-/-^ mice confirmed the specificity of our approach. Mann2-DTPA-Gd MRI can track the changes of CD206^+^ macrophages at different stages of wound healing, correlating well with flow cytometry data using another anti-inflammatory macrophage marker (arginase-1). The specificity and efficacy of Mann2-DPTA-Gd were further validated in experimental glioma, in which Mann2-DTPA-Gd imaging detected CD206^+^ tumor-associated macrophages (TAMs), demonstrated significantly decreased signals in *Mrc1*^+/-^ mice and *Mrc1*^-/-^ mice, and tracked treatment changes in D-mannose-treated *Mrc1*^+/+^ mice. Furthermore, Mann2-DTPA-Gd can report microglia/macrophages and correlate with histology in stroke. The more Gd-stable agent MannGdFish demonstrated similar efficacy as Mann2-DTPA-Gd *in vivo* with favorable biodistribution and pharmacokinetics.

**Conclusion:** We have developed a fluorescent agent (MR2-cy5) and MRI agents (Mann2-DTPA-Gd and MannGdFish) with two mannose moieties that are highly specific to CD206 and can track CD206^+^ macrophages in disease models of wound healing, tumor, and neurological disease. Importantly, MannGdFish, with its high specificity, stability, favorable biodistribution, and pharmacokinetics, is a promising translational candidate to noninvasively monitor CD206^+^ macrophages in repair/regeneration and tumors in patients. In addition, with the specific binding motif to CD206, other imaging modalities and therapeutic agents could also be introduced for theranostic applications.

## Introduction

While aberrant innate immune response causes damage, the innate immune response can also bring about repair and healing. Macrophages are key drivers of these responses. Different subsets of activated macrophages may play distinct roles and can adopt different phenotypes in response to microenvironmental stimuli 1 in various pathophysiological processes such as wound healing 2, cancer 3, myocardial infarction (MI) 4, and neurological diseases 5, 6, among others. Reprogramming to shift from a damaging phenotype to a reparative phenotype is a promising strategy in stroke 7, MI 8, and especially in tumor-associated macrophages (TAMs)-targeted immunotherapies 3, 9-11. Thus, agents that can specifically target specific types of macrophages are highly desirable.

In particular, elevated expression of CD206 on activated macrophages has been found in various pathophysiological conditions 4, 12-14 and these CD206^+^ cells have become an emerging therapeutic target 13, 15, 16. CD206, encoded by the gene *Mrc1*, is a 175 kDa transmembrane protein that recognizes and mediates endocytosis of pathogens by binding to glycoproteins terminated with mannose, fucose, or N-acetyl-glucosamine 17-19. Imaging agents targeting CD206^+^ macrophages have been reported using either D-mannose-based agents 20-23 or CD206-specific antibody/nanobody-based SPECT/PET imaging 24-26. However, antibody/nanobody-based SPECT/PET imaging has radiation exposure concerns that limit the frequency of imaging, and other single mannose-containing agents have issues with specificity since it has a relatively low binding affinity to CD206 compared to its mannoside analogs 27, 28. However, no study has been conducted to evaluate the potency of these mannosides as imaging agents in part due to the challenges in synthesizing these carbohydrate-containing agents, especially for MRI, which has excellent spatial resolution and soft tissue contrast but a higher amount of the agents is required compared to PET/SPECT or fluorescent agents. In this study, we report the development of a multi-mannose-based approach to specifically target CD206^+^ macrophages by designing CD206^+^-targeting fluorescent and MRI agents and validating these agents in animal models of wound healing, glioma, and stroke.

## Methods

All chemicals were obtained from Sigma Chemical Co. unless otherwise stated. D-mannosamine hydrochloride was purchased from Biosynth Carbosynth (United Kindom), DOTA-GA anhydride from CheMatech (Dijon, France), Z-Ser-OH from Ambeed (Illinois, USA) and 1,2,3,4,6-penta-O-benzoyl-⍺-D-mannopyranose from BOC Science (New York, USA). ^1^HNMR and ^13^CNMR were recorded with a JEOL 11.7 T NMR system equipped with a 5 mm broadband probe. Flash chromatography was performed with Combiflash (Telydyne ISCO CombiFlash, CA) with UV detection at 220 and 254 nm. High-resolution mass spectrometry was performed with Thermo Scientific^TM^ Q-Exactive Plus Ultimate 3000 HPLC flow injection analysis. Inductively coupled plasma mass spectrometry (ICP-MS) was conducted on an Agilent 8800-QQQ system. Flow cytometry data were acquired with LSRII flow cytometer (BD Bioscience). All animal experiments were carried out in compliance with the National Institutes of Health's 'Guide for the Care and Use of Laboratory Animals' and were approved by and in compliance with the Institutional Animal Care and Use Committee at Massachusetts General Hospital. All mice were allowed to acclimate for one week prior to the start of the experiments.

### Chemical synthesis and characterization

#### Synthesis of MR1-cy5

To a solution of D-mannosamine **1** (5 mg) in DMSO (1 mL) was added triethylamine (10 μL) and stirred for 20 min, followed by a solution of compound **2** Cy5-NHS (10 mg, 1 equiv.) in DMSO (1 mL). The reaction was stirred for another 1 hour at room temperature. The reaction was filtered to remove the solid and subjected to the HPLC separation to provide MR1-cy5 (3.7 mg, 35%). High-resolution MS: 840.2803 (M+Na, cal. 840.2806).

#### Synthesis of Mann2-DTPA-Gd

To a solution of D-mannosamine **1** (472 mg, 2.2 equiv.) in DMSO (6 mL) was added triethylamine (700 μL, 5.0 equiv.) and stirred for 30 min, then DTPA dianhydride **3** (318 mg, 1.0 equiv.) was added portionwise and stirred for another 3 h. The reaction underwent reversed column to give compound Mann2-DTPA **3** (460 mg, 64%). LCMS found m/z: 716.2 (M+H). A solution of the above compound **4** (286 mg, 1.0 equiv.) in water was mixed with a solution of GdCl_3_ (148 mg, 1.0 equiv.) in sodium ascorbate butter (pH 5.5) and stirred for 1 h. The reaction was filtered and HPLC separation gave the desired compound Mann2-DTPA-Gd (87 mg, 50%). High-resolution MS: 871.1849 (M+H, cal. 871.1845).

#### Synthesis of MR1-cy5

#### Synthesis of Mann2-DTPA-Gd

#### Synthesis of MR2-cy5

Synthesis of compound **7**. Compound **5** (360 mg, 1.0 equiv.), compound **6** obtained from bromination of 1,2,3,4,6-penta-O-benzoyl-alpha-D-mannopyranose 29 (2.32 g, 2.2 equiv.) and activated 4Å molecular sieves (1.0 g) in dichloromethane (12 mL) was stirred for 10 min. Then silver triflate (946 mg, 2.3 equiv.) was added to the reaction mixture at 0 °C. The reaction was warmed to room temperature and stirred for another 2 h to complete. Saturated NaHCO_3_ (5 mL) was added to the reaction and filtered. The filtrate was washed with brine, concentrated, dried, and underwent flash chromatography (gradient of hexane/ethyl acetate: 100% to 40%) to give the desired compound **7** (1.43 g, 65%) as a white powder. ^1^H NMR (500 MHz, CDCl_3_) δ: 8.10 (d, 4 x 1H, J = 8 Hz), 7.99 (d, 4 x 1H, J = 7.5 Hz), 7.93 (d, 4 x 1H, J = 8 Hz), 7.80 (d, 2 x 1H, J = 7.0 Hz), 7.76 (d, 2 x 1H, J = 7.5 Hz), 7.59-7.52 (m, 4H), 7.45-7.30 (m, 17H), 7.29-7.20 (m, 9H), 6.15 (t, 2 x 1H, J = 10 Hz), 5.90 (dd, 2 x 1H, J1 = 10 Hz, J2 = 3 Hz), 5.75 (d, 2 x 1H, J = 12 Hz), 5.48 (d, 1H), 5.22-5.14 (m, 4H), 4.76 (d, 2 x 0.5H, J = 11.5 Hz), 4.69 (m, 2 x 0.5H, J = 10.5 Hz), 4.57 (d, 2 x 0.5H, J = 12 Hz), 4.54-4.48 (m, 3H), 4.29 (b, 1H), 4.10-4.07 (m, 1H), 3.94 (d, 2 x 0.5H, J = 11.5 Hz), 3.79-3.76 (m, 1H). ^13^C NMR (125 MHz, CDCl_3_) δ: 166.11, 166.08, 165.44, 165.39, 165.23, 155.96, 136.15, 133.36, 133.32, 133.25, 133.07, 133.01, 132.96, 129.83,129.75, 129.71, 129.68, 129.20, 129.16, 128.89, 128.85, 128.82, 128.52, 128.48, 128.41, 128.40, 128.33, 128.29, 128.21, 98.26, 98.08, 70.16, 70.07, 69.93, 69.32, 69.25, 67.59, 67.14, 66.98, 66.59, 62.70, 49.99. LCMS found m/z: 1382.4 (M+H).

Synthesis of compound **8**. To a solution of compound **7** (1.1 g, 1.0 equiv.) in ethyl acetate and ethanol (v/v: 1/1, 5 mL) was added Pd/C (palladium on carbon, 110 mg) and stirred vigorously with a hydrogen balloon overnight. The reaction mixture was filtered and concentrated to provide compound **8** without further purification. ^1^H NMR (500 MHz, CDCl_3_) δ 8.10 (d, 2 x 1H, J = 3.5 Hz), 8.09 (d, 2 x 1H, J = 3.5 Hz), 7.95 (d, 2 x 1H, J = 7.5 Hz), 7.91-7.89 (m, 6H), 7.73 (d, 2 x 1H, J = 7 Hz), 7.69 (d, 2 x 1H, J = 7.5 Hz), 7.53-7.46 (m, 4H), 7.40-7.35 (m, 6H), 7.34-7.29 (m, 4H), 7.27-7.22 (m, 4H), 7.19-7.16 (m, 4H), 7.14-7.10 (m, 2H), 6.21 (t, 2 x 1H), 5.96 (dd, 2 x 1H, J1 = 10 Hz, J2 = 3 Hz), 5.89 (m, 2H), 5.29 (d, 2 x 1H, J = 19.5 Hz), 4.83-4.80 (m, 2H), 4.64 (d, 2 x 1H, J = 10 Hz), 4.55-4.50 (m, 2H), 4.40 (d, 1H, J = 7.5 Hz), 4.34 (dd, 1H, J1 = 10 Hz, J2 = 4 Hz), 4.17-5.10 (m, 3H), 3.33 (b, 2H). ^13^C NMR (125 MHz, CDCl_3_) δ 166.11, 165.99, 165.92, 165.50, 165.30, 165.23, 133.29, 133.16, 133.08, 132.95, 129.85, 129.77, 129.73, 129.66, 129.00, 128.88, 128.85, 128.73, 128.68, 128.41, 128.33, 128.24, 128.16, 98.37, 98.31, 70.76, 70.56, 69.95, 69.73, 69.46, 66.02, 65.78, 65.48, 62.45, 62.36, 50.97. LCMS found m/z: 1248.2 (M+H).

Synthesis of MR2-cy5. To a solution of compound **8** (20 mg, 1.2 equiv.) in DMSO (0.6 mL) was added triethylamine (10 μL) followed by a solution cy5-SE (10 mg, 1.0 equiv.) in DMSO (0.6 mL). The reaction was stirred at room temperature for 2 h and monitored by LC-MS to give the major product **9**. Water (10 mL) was added to the reaction and the precipitate was filtered, washed and put to the next step without further purification. To a suspension of the above precipitate in methanol (10 mL) was added a solution of sodium methoxide (25% weight in methanol, 1.1 equiv. to compound **8**). The reaction was stirred at room temperature for 4 h. Minimum water was added to the reaction to dissolve any suspension, and the solvent was removed under vacuum. The remainder was subjected to HPLC to afford the desired compound MR2-cy5 in the yield of 31% for two steps. High-resolution MS: 1054.3878 (M+H, cal. 1054.3883).

#### Synthesis of MannGdFish

Synthesis of compound **11**. To a solution of compound **8** (374 mg, 1.0 equiv.) in DMSO (3 mL) was added N, N-diisopropylethylamine (130 μL, 2.5 equiv.) and subsequent compound **10** (138 mg, 1.0 equiv.). The reaction was stirred at 70 °C overnight. The reaction underwent reversed phase column (gradient of water/acetonitrile: 95/5 to 0/100) to give compound **11** (363 mg) as a white solid in the yield of 71%. ^1^H NMR (500 MHz, DMSO) δ: 8.76 (b, 1H), 8.05 (d, 4 x 1H, J = 6Hz), 7.90 (d, 4 x 1H, J = 6.5 Hz), 7.85 (d, 4 x 1H, J = 7 Hz), 7.70 (b, 4 x 1H), 7.64-7.44 (m, 16H), 7.37-7.23 (m, 8H), 6.02 (t, 2 x 1H, J = 10 Hz), 5.85-5.70 (m, 4H), 5.36-5.32 (m, 2H), 4.73-4.57 (m, 6H), 4.41 (b, 1H), 4.03-4.01 (m, 2H), 3.90-3.77 (m, 3H), 3.53-3.36 (m, 12H), 3.04-2.82 (m, 10H), 2.69-2.63 (m, 2H), 2.39-2.35 (m, 1H), 1.98 (b, 1H), 1.81 (m, 1H). ^13^C NMR (125 MHz, DMSO) δ 172.80, 172.28, 170.52, 170.44, 165.28, 164.96, 164.70, 164.60, 133.94, 133.63, 133.55, 129.55, 129.29, 129.18, 128.93, 128.86, 128.63, 97.45, 97.36, 97.27, 97.20, 70.47, 69.91, 69.85, 68.44, 68.40, 67.04, 65.88, 62.62, 62.08, 55.37, 55.28, 54.37, 51.05, 50.94, 50.59, 50.40, 49.71, 49.63, 47.92, 47.80, 46.95, 32.58, 32.46. LCMS found m/z: 1708.3 (M+H).

#### Synthesis of MR2-cy5

#### Synthesis of MannGdFish

Synthesis of MannGdFish. To a solution of compound **11** (256 mg, 1.0 equiv.) in methanol was added sodium methoxide (25% weight in methanol, 1.2 equiv.) and stirred for 3 h at room temperature. The reaction was adjusted to pH 7 with 1M HCl, concentrated to remove methanol, and the liquid phase was washed with ethyl acetate and lyophilized to give the deprotected compound without further purification. LCMS found m/z: 874.2 (M+H). A solution of the above compound was added to the solution of GdCl_3_ (56 mg, 1.05 equiv.) in sodium acetate buffer (pH 5.5, 4 mL). The reaction was stirred at room temperature for 1 h. The reaction underwent reversed phase column to give the desired compound MannGdFish (68 mg, 45% for two steps). High-resolution MS: 1029.2790 (M+H, cal. 1029.2791).

### *In vitro* relaxivity of Mann2-DTPA-Gd/MannGdFish

Relaxation time (T1) of Mann2-DTPA-Gd/MannGdFish at concentrations of 0.1, 0.2, 0.3, 0.5, 0.6, 0.75, and 1 mM in PBS was measured on the Bruker Minispect (Bruker Analytics, MA) at 0.47 T (20 MHz) at 40 °C. The slope value of the linear function of 1/T1(s) to their corresponding concentrations is defined as the relaxivity (n =3 for each concentration).

### Pro-inflammatory (CD206^-^) and anti-inflammatory (CD206^+^) polarized differentiation

Raw 264.7 cells (Passage 8 from the cell core of center for Systems Biology at Massachusetts General Hospital, Boston) in 10 mL petri dishes were cultured with Dulbecco's modified eagle medium with high glucose (DMEM, Thermo Fisher Scientific, NY, USA) containing 10% of fetal bovine serum (FBS, Sigma-Aldrich, MO, USA) and 0.5% streptomycin/penicillin (Cellgro) until reaching 80% of confluency. Then differentiating media containing IFN-gamma (100 ng/mL the above medium) for CD206^-^ polarization and IL-4 (100 ng/mL) for CD206^+^ polarization were added to the dishes, respectively. After 24 h, these differentiated macrophages were ready for the following experiments.

### Fluorescence imaging

IFN-gamma induced D206^-^ and IL-4 induced CD206^+^ macrophages were incubated with 1/1000 dilution of MR1-cy5/MR2-cy5 stock solution (10 mM in DMSO) for 1h at 37 °C and counter-stained with DAPI (4',6-diamidino-2-phenylindole, Invitrogen). The cells were washed with PBS and fluorescence imaging was captured with a digital microscope (Nikon Eclipse TE2000-U).

### ICP-MS

Differentiated CD206^-^ and CD206^+^ macrophages and HEK293 cells were incubated with 1 mM of Mann2-DTPA-Gd for 1h at 4 °C. After washing with PBS (x 3), cells were collected, digested with nitric acid (70%, 200 μL) overnight and subjected to an Agilent 8800-QQQ system (Agilent, MA, USA) to detect the amount of Gd.

### Flow cytometry

Differentiated CD206^-^ and CD206^+^ macrophages were collected, centrifuged, and resuspended in FACS buffer. For surface and intracellular staining anti-CD16/CD32, anti-CD11B APC/CY7, anti-CD86 Pacific Blue, and anti-CD206 FITC were obtained from Biolegend (San Diego, CA). Cells were incubated first with anti-CD16/CD32 to block Fc binding site for 20 minutes then washed (x3). Cells were then incubated with antibodies against surface markers for 20 min at 4 °C in the dark. For intracellular staining cells were then fixed and permeabilized using 1X Fix/Perm solution (BD Bioscience), washed in 1X permeabilization buffer (BD Bioscience), stained with anti-CD206 FITC for 30 minutes at 4 °C in the dark. Cells were subsequently washed and resuspended in FCS buffer. Anti-inflammatory cells were identified as CD11B^+^, CD206^+^ cells. Data were acquired on LSRII flow cytometer (BD Bioscience) and analyze with BD FlowJo software (10.4).

### Subcutaneous wound healing

#### Wound healing model induction

Seven- to ten-week-old C57BL/6J female mice (The Jackson Laboratories, ME), B6.129S4-Arg1^tm1.1Lky^/J mice (Jackson laboratory), and mannose-receptor deficient mice (Jackson laboratory) were used in this study. A circular outline on the back of a mouse was made with a 4 mm biopsy punch (Kai Medical, Japan) under anesthesia using isoflurane. Then a full-thickness excisional wound was generated that extended through the subcutaneous tissue. A silicone splint was glued over the wound, anchored with 6-0 nylon sutures, and covered with a transparent occlusive dressing (OpSite). Xylazine/norepinephrine (100 μL) were intraperitoneally injected twice a day for two days and the mice were monitored daily.

#### Histological analysis

Wound tissues from C57BL/6J mice were excised on days 1, 4, and 7 and fixed with 4% formaldehyde for 24 hours at room temperature. The fixed tissues were embedded with paraffin and sectioned to 5 µm thickness, then hematoxylin and eosin (H&E) staining was performed following the manufacture instruction (Vector lab). Images were captured with the Nikon DS-Ri2 model microscope connected to Prime BSI Express.

#### Flow cytometry

To isolate different macrophage subsets, wound tissues from B6.129S4-Arg1^tm1.1Lky^/J mice were excised on days 1, 4, and 7, cut into small pieces and incubated for 1 hour at 37 °C in HBSS containing 0.7 mg/mL collagenase D (Milipore Sigma). Then the skin tissues were passed through 70 μm (BD Biosciences, San Jose, CA) strainer, and single cells were isolated from 44/67 Percoll (GE Healthcare, Boston, MA) gradient interface. For staining anti-CD16/CD32, anti-CD11B APC/CY7, anti-CD45 Pacific blue and anti-CD86 APC were obtained from Biolegend (San Diego, CA). Cells were then stained as described above. Reparative/anti-inflammatory macrophages were identified as CD11B^+^, CD45^+^, Arg1^YFP+^, CD86^-^ cells and damaging/pro-inflammatory macrophages were identified as CD11B^+^, CD45^+^, CD86^+^, and Arg1^YFP-^ cells. Data were acquired and analyzed as described.

### Mouse model of glioma

CT-2A-luc tumor cell culture was followed the previously published protocol 30. Briefly, the cells were incubated at 37 °C with humidified air containing 5% CO_2_. Monolayer CT-2A-luc cells were cultured in DMEM supplemented with 10% FBS and 1% penicillin-streptomycin. To generate neurospheres, CT-2A monolayer cells were enzymatically dissociated by accutase (Stem Cell Technology, San Diego) and seeded in 25 cm^2^ culture dishes at the cell concentration of 1 × 10^5^ cells/mL in serum-free medium, composed of advanced DMEM/F12 medium (Life Technologies, Carlsbad, CA) with L-glutamine (2 mM; Cellgro, Manassas, VA), 1% N2 supplement (Life Technologies), 1% penicillin-streptomycin, recombinant EGF (20 ng/mL; R&D Systems, Minneapolis, MN), and recombinant FGF2 (20 ng/mL; Peprotech, East Windsor, NJ). After 10-11 days, the neurosphere CT-2A-luc (NS/CT-2A-luc) cells were collected, enzymatically dissociated with accutase, and prepared for intracranial injection.

Eight- to nine-week-old C57BL/6J female and male mice (*Mrc1*^+/+^), *Mrc1*^+/-^, and *Mrc1*^-/-^ mice were used in this experiment (Jackson laboratory, ME). Dissociated NS/CT-2A-luc cells (7-8 × 10^4^) were implanted stereotactically into the brain (2.5 mm lateral and 1 mm anterior to Bregma and 3 mm deep) to generate an orthotopic intracranial tumor 31. The mice then were monitored daily for signs of discomfort or neurological symptoms. For the CD206 competition study, D-mannose (450 mg/kg) was injected intraperitoneally to the wildtype glioma mice 30 mins before Mann2-DTPA-Gd administration. MR imaging was carried out between the third to fourth week after implantation.

### Mouse model of stroke

Transient focal cerebral ischemia was induced in 2-3 months old male C57BL/6J mice (n = 3). Mice were anesthetized with 2% isofluorane. A rectal temperature probe was inserted to monitor and maintain a constant animal core temperature of 37 ± 0.5 °C using a temperature controller (TC-1000, CWE INC, Ardmore, PA) and mice was induced by MCAO as described by Liu et al 32. Briefly, an 8-0 nylon monofilament suture (ETHICON LLC, Puerto Rico, USA) coated with silicone rubber (Heraeus Kulzer LLC, South Bend, IN) and hardener was inserted into the left internal carotid artery and advanced approximately 10 mm distal to the carotid bifurcation to occlude the origin of the middle cerebral artery. The thread was carefully withdrawn 30 min after MCAO to induce I/R injury.

### MR imaging

Mice with subcutaneous wounds were imaged on days 1, 4, and 7 longitudinally or on day 7. Mice with glioma were imaged between the third and fourth week. Mice with stroke were imaged on day 11 post-stroke. A T2-weighted sequence was performed for glioma and stroke mice before probe administration. All the mice were imaged pre- and at 0, 15, 30, 45, and 60 min after 0.3 mmol/kg of Mann2-DTPA-Gd/MannGdFish/DOTA-Gd was administered intravenously through a tail vein using serial T1 rapid acquisition with relaxation enhancement (RARE) sequence (TR: 935,77 ms, TE: 13.59 ms, averages: 12, rare factor: 4, 256 x 256 x 48 matrix size, 0.156 x 0.156 x 1 mm^3^ voxel size) with chemical fat suppression using a Hermitian pulse shape with an 8.253 ms pulse and 701.19 Hz bandwidth 3.5 ppm down from the water peak and respiratory gating on a 4.7 T small animal MR scanner (Bruker, Cambridge, MA) with a dedicated mouse head coil. Regions of interest (ROI) were drawn manually, and the contrast-to-noise ratios (CNRs) were calculated by an experienced radiologist blinded to the identity of the imaging agent used and the strain of mice (n = 3 mice for each group). CNR = (SI_tumor_ - SI_normal brain_)/SD_background_. The CNR at a given time point was corrected by the pre-contrast CNR (i.e., CNR_post-contrast_ - CNR_pre-contrast_).

### Immunofluorescent staining

Fresh-frozen brains were cut in serial cross sections (10-12 µm thick). The sections were fixed with 4% PFA for 15-20 minutes at room temperature (RT). The slides were incubated in an avidin/Biotin Blocking Kit (Abcam, Cat# ab64212) following manufacturer instructions. The slides were incubated with the primary antibody anti-CD206 biotin-conjugated (1:100, Thermo Fisher, Cat# MA5-16869) overnight at 4 °C (with 0.3% triton x-100). On the second day, slides were rinsed with PBS for 3 times and then incubated with Streptavidin, Dylight 488 (1:300, Thermo Fisher, Cat# 21832), and MR2-cy5 (1:250, 10 mM in DMSO) at RT for 1 hour. The sections were then again washed 3 times with PBS and then the sections were mounted in an antifade mounting medium (Vectashield, Cat# zj0808) and DAPI. Images were captured with the Nikon DS-Ri2 model microscope connected to Prime BSI Express. Colocalization analysis was performed using a JACoP (Just Another Colocalization Plugin) in ImageJ.

### Cytotoxicity of MannGdFish

The cytotoxicity of MannGdFish was evaluated using RAW264.7 cells (passage 8 from the cell core at the Center for Systems Biology at the Massachusetts General Hospital, Boston) with an MTT assay as described previously (n = 3) 33.

### Biodistribution, retention and blood half-life of MannGdFish

6-10 weeks old of C57BL/6J mice were administered with MannGdFish intravenously 24 h after wound injury. At 3 h and on day 7, mice were sacrificed, and major organs, including wound skins and the normal skins, were harvested (n =3 for each time point). Blood samples were collected before and at different time points after the administration of MannGdFish (n = 6) and centrifuged to obtain the plasma. The samples were weighed, treated with nitric acid, and subjected to the ICP-MS to determine the contents of gadolinium as described above.

### Statistical analysis

All numeric data were first analyzed for normality using the Shapiro-Wilk normality test with significance to determine the appropriate parametric or nonparametric test to use. One-way ANOVA and two-way ANOVA were used for the data analysis. All statistical analyses were performed with GraphPad Prism version 10 (GraphPad Software, La Jolla California) and data are shown with mean ± SEM. A p-value < 0.05 was considered statistically significant.

## Results

### Agent development

**Scheme 1:**
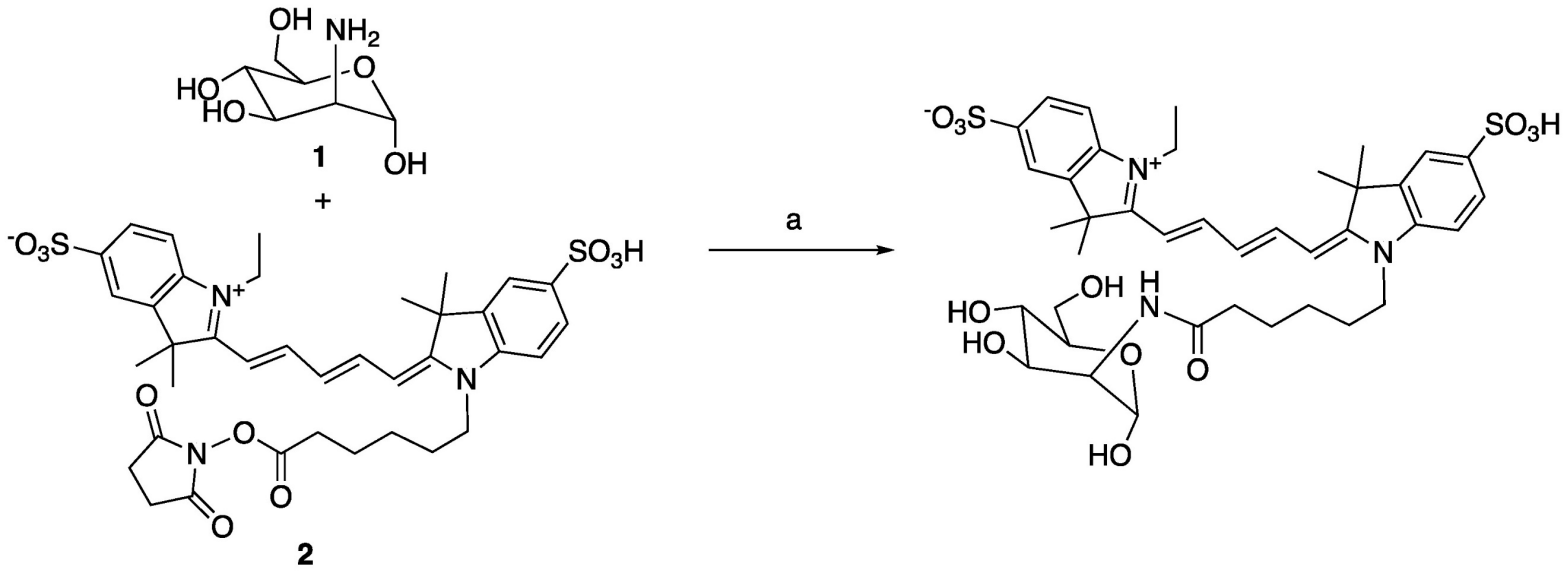
a) NEt_3_, DMSO, rt, 35%.

**Scheme 2:**
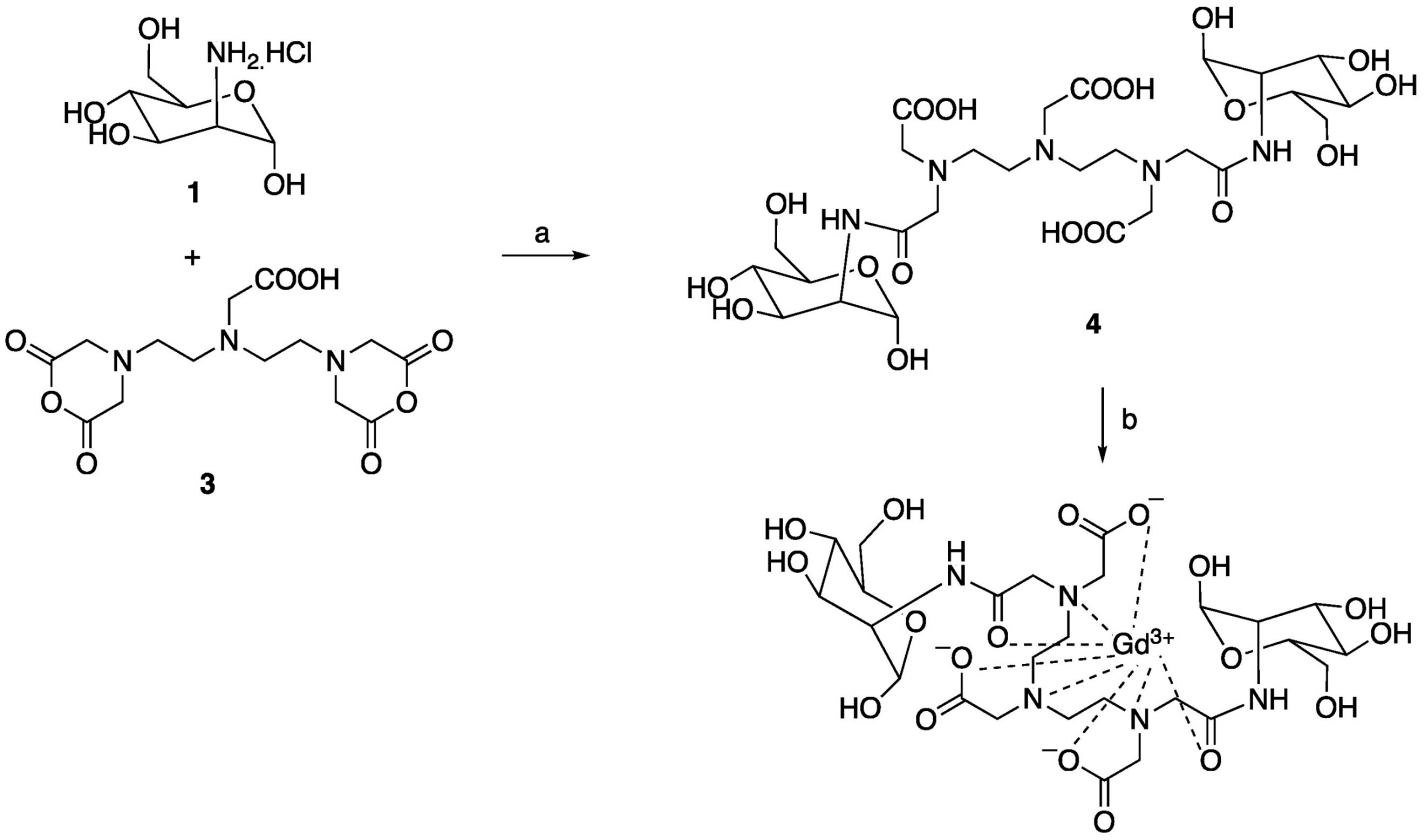
a) NEt_3_, DMSO, rt; b) GdCl_3_, sodium citrate buffer, pH 5.5. The total yield for two step synthesis of Mann2-DTPA-Gd was 32%.

**Scheme 3:**
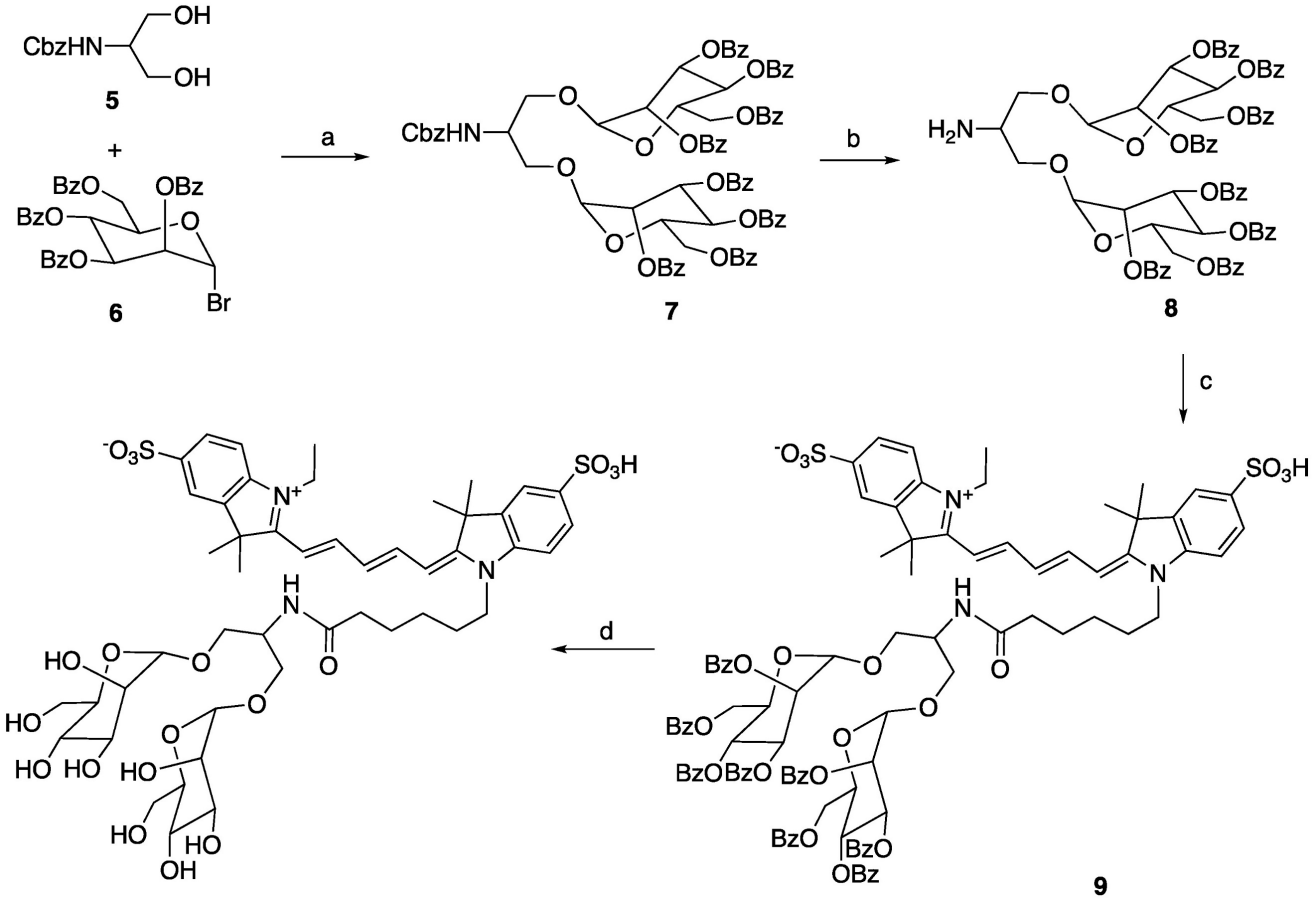
a) AgOTf, 4Å molecular sieves, rt, 65%; b) H_2_, Pd/C, MeOH, rt, quantitative; c) Cy5-NHS ester, NEt_3_, DMSO; d) NaOMe/MeOH, 31% for the last two steps.

**Figure 1:**
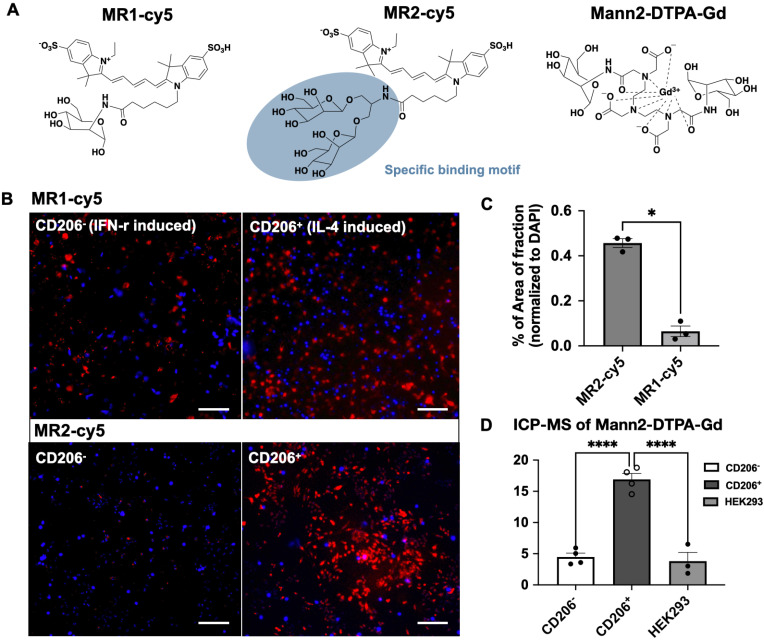
** Structure of fluorescent and MRI agents and *in vitro* validation. A)** Structures of MR1-cy5, MR2-cy5, and Mann2-DTPA-Gd.** B)** Fluorescence imaging of MR1-cy5 incubated with differentiated macrophages at 37 °C showed low specificity for CD206^+^ macrophages, while MR2-cy5 showed a much higher signal for CD206^+^ macrophages than that for CD206^-^ macrophages, indicating specificity for CD206^+^ macrophages. **C)** Quantification of fluorescence signals showed a much higher signal intensity of MR2-cy5 to CD206^+^ macrophages compared to that of MR1-cy5 (one-way Mann Whitney test. *, p = 0.05, n = 3. Images: 10x, scale: 100 μm).** D)** The amount of gadolinium detected by ICP-MS on CD206^+^ macrophages was much higher than that on CD206^-^ macrophages and HEK293 cells (as non-specific control) when incubated with Mann2-DTPA-Gd (one-way ANOVA. n = 4 for CD206^-^ and CD206^+^ macrophages and n = 3 for HEK293 cells. ****, p < 0.0001 for both CD206^-^ vs. CD206^+^, and HEK293 vs. CD206^+^).

Since the binding affinity of the lysine-based mannoside containing two mannose units is about 100 times higher than that of D-mannose (18-23 µM vs. 5.5 mM) 28, 34, we first developed a series of MRI and fluorescent imaging agents containing one or two mannose moieties to evaluate their efficacy to CD206 and CD206^+^ macrophages (**Figure 1A**). MR1-cy5 containing a single mannose was synthesized by coupling D-mannosamine with cy5-NHS-ester (Methods **Scheme 1**). A prototype MRI agent containing two mannose moieties, Mann2-DTPA-Gd, was also synthesized by coupling D-mannosamine with DTPA anhydride under basic conditions followed by chelating with GdCl_3_ with a total yield of 32% (Methods **Scheme 2).**The synthesis of MR2-cy5 containing two mannose moieties, however, turned out to be more challenging. After trying different scaffold linkers and protective groups to the hydroxy group of the mannose unit, we made compound **8** as the key intermediate not only for the synthesis of MR2-cy5 (Methods **Scheme 3**) but also for the synthesis of MannGdFish (see below). Intermediate **8** was coupled with cy5-NHS ester followed by deprotection to provide MR2-cy5 (Methods **Scheme 3**). The detailed synthetic routes and characterization of these agents are described in the Experimental Section and **Figures S1-S3**. The relaxivity (r_1_) of MR2-DTPA-Gd was 3.6 mM^-1^s^-1^ determined by using a Bruker Minispect (Bruker Analytics, MA) at 0.47 T (20 MHz) in PBS at 40 °C (**Figure S4**).

### *In vitro* validation of specificity to the mannose receptor (CD206)

We validated the specificity of MR1-cy5, MR2-cy5, and Mann2-DTPA-Gd for CD206 in cellular assays using differentiated macrophages by IFN-gamma and IL-4 from Raw 264.7 cells 35, respectively. The differentiation was confirmed by flow cytometry with a much higher percentage of CD206^+^ cells in IL-4-induced macrophages than in IFN-gamma-induced macrophages (**Figures S5** and** S6**). However, when incubated with MR1-cy5 containing only a single mannose moiety, fluorescent imaging showed similar signals from both types of activated macrophages (**Figure 1B**), revealing low specificity of MR1-cy5 for CD206^+^ macrophages. In contrast, fluorescent imaging from MR2-cy5, which contains two mannose moieties in a clustered structure, demonstrated a markedly higher signal only in IL-4-induced CD206^+^ macrophages compared to that in IFN-gamma-induced CD206^-^ macrophages incubated with MR2-cy5 and IL-4-induced CD206^+^ macrophages incubated with MR1-cy5 (**Figure 1B** and** 1C**). We then incubated the MRI agent Mann2-DTPA-Gd with both activated macrophages and human embryonic kidney cells (HEK293, a non-specific binding control). As expected, the amount of gadolinium in IL-4-induced CD206^+^ macrophages was markedly higher than that in IFN-gamma-induced CD206^-^ macrophages and HEK293 cells (**Figure 1D**). Together these results confirmed the higher specificity of these agents containing two mannose units for CD206^+^ cells.

### MR imaging of Mann2-DTPA-Gd in a mouse model of subcutaneous wound healing

**Figure 2:**
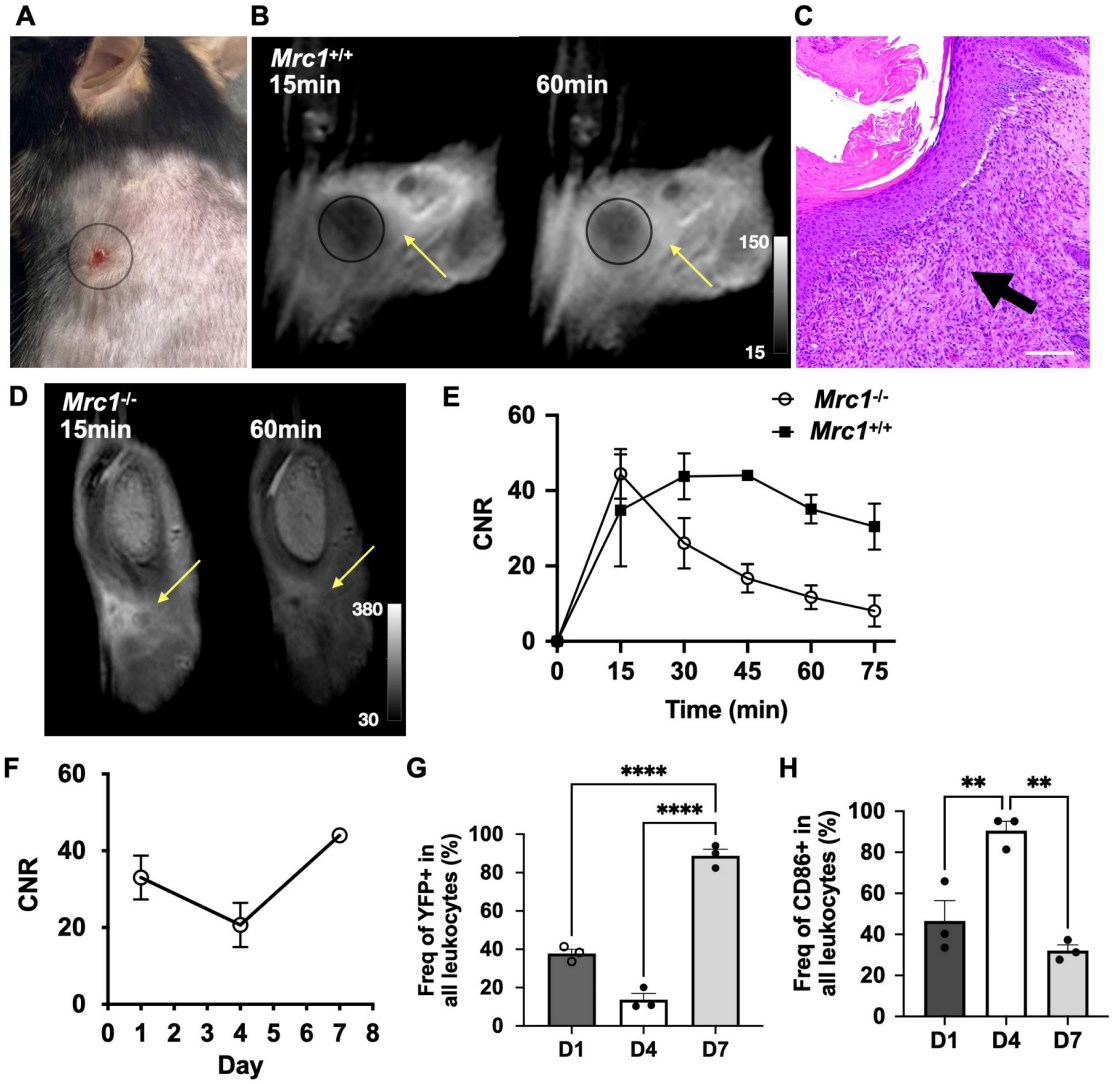
** MR imaging of Mann2-DTPA-Gd in a mouse model of subcutaneous wound healing. A)** Subcutaneous wound on day 7. **B)** MR images of Mann2-DTPA-Gd at 15 min and 60 min in *Mrc1*^+/+^ mice. **C)** H&E staining of wound tissues on day 7 correlates well with MR imaging. Arrow indicated the filtrated immune cells around the wound areas. Images: 10x; scale: 100 μm. **D)** MR images of Mann2-DTPA-Gd at 15 min and 60 min in *Mrc1*^-/-^ mice. **E**) CNR of *Mrc1*^-/-^ mice decreased significantly after 15 min post-injection compared to that in *Mrc1*^+/+^ mice on day 7 (n = 3, Mann2-DTPA-Gd administration at 0.3 mmol/kg mouse). **F)** Longitudinal MR imaging of Mann2-DTPA-Gd at 60 min post-injection showed that CNR on day 7 was much higher compared to that on days 1 and 4. **G)** Percentage of YFP^+^ cells (arginase-1-positive cells, a marker for anti-inflammatory/reparative cells) on day 7 was significantly higher than that on day 1 and day 4 (n = 3 per group, One-way ANOVA. ****, p <0.0001 for both days 1 vs. 7 and days 4 vs. 7) from flow cytometry. **H)** Percentage of CD86^+^ cells (a marker for proinflammatory cells) on day 4 was significantly higher than that on day 1 and day 7 (n = 3, One-way ANOVA, **, p = 0.005 and p = 0.001, respectively, for days 1 vs. 4 and days 7 vs. 4). CNR = contrast-to-noise ratio.

We next evaluated the specificity and ability of Mann2-DTPA-Gd to track CD206^+^ macrophages in a mouse model of wound healing since this model has well-defined inflammatory and reparative stages where activated macrophages play distinctive roles 2. A circular subcutaneous wound was made on the upper back of the mice and monitored on days 1, 4, and 7 (**Figures S7 and S8**). MRI of Mann2-DTPA-Gd was performed at the reparative stage when CD206^+^ macrophages predominate (peaking around day 7). In *Mrc1*^+/+^ mice on day 7 after wound induction (**Figure 2A**), there was increased contrast enhancement at 60 min compared to 15 min after Mann2-DTPA-Gd administration (**Figure 2B**). The H&E staining confirmed the infiltration of immune cells around the wound areas on day 7 (**Figure 2C**). On the other hand, in *Mrc1*^-/-^ mice, the signal intensity increased during the first 15 min post-injection due to the wash-in of the agent, then decreased drastically (washout) over time (**Figure 2D** and** 2E**), while the contrast-to-noise ratio (CNR) of Mann2-DTPA-Gd increased over time until 45 min post-injection prior to slowly decreasing (**Figure 2E**), confirming the *in vivo* binding and specificity of Mann2-DTPA-Gd for CD206.

Having confirmed the specificity of Mann2-DTPA-Gd, we performed a longitudinal study to track changes in CD206^+^ macrophages in wound healing. Using the CNR at the 60 min time point, we found that some CD206^+^ cells were present on day 1 after wound induction which decreased on day 4 prior to increasing again on day 7 (**Figure 2F**). There was approximately 2- to 3-fold higher CNR from 45-75 min post-injection on day 7 compared to that on day 4 (**Figure S9**), revealing the evolution of CD206^+^ macrophages at different stages of healing. To validate the MRI data, we next performed a flow cytometric study to differentiate pro-inflammatory and anti-inflammatory macrophages in the same model using transgenic YFP-labeled arginase-1 (YARG) mice on days 1, 4, and 7 36. Arginase-1 (Arg1) is another marker for anti-inflammatory/reparative macrophages 3. The gating strategy for flow cytometric analysis is shown in **Figure S10**. As expected, the flow cytometric data (**Figure 2G**) mirrored the results from Mann2-DTPA-Gd MRI (**Figure 2F**). To ensure that Mann2-DTPA-Gd imaging was not detecting pro-inflammatory macrophages, we also assessed the macrophages by flow cytometry using CD86 as a pro-inflammatory marker 3, which showed an opposite trend (peaking on day 4) compared to what was detected by Mann2-DTPA-Gd (**Figure 2H**). Taken together, these data demonstrated that MR imaging of Mann2-DTPA-Gd is capable of noninvasive mapping and tracking of the dynamic changes of CD206^+^ anti-inflammatory/reparative macrophages in wound healing.

### MR imaging of experimental glioma with Mann2-DTPA-Gd

**Figure 3:**
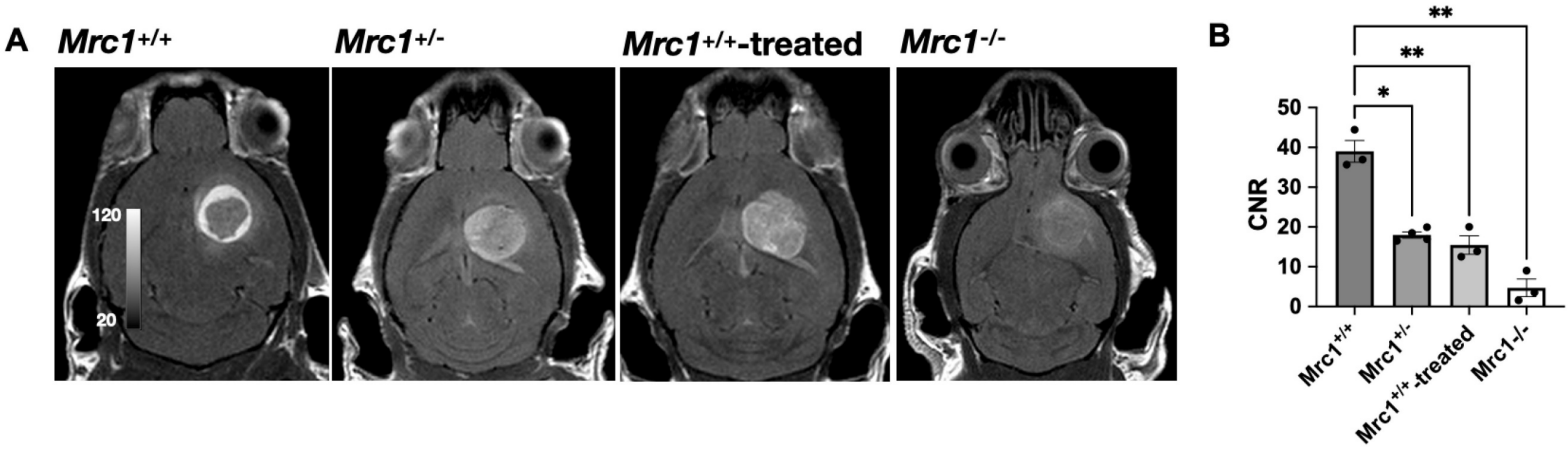
** MR imaging of glioma with Mann2-DTPA-Gd. A)** Representative images of glioma in *Mrc1*^+/+^, *Mrc1*^+/-^, *Mrc1^-/-^*, and *Mrc1*^+/+^ mice treated with D-mannose at 60 min post-injection. **B)** Contrast-to-noise ratios (CNRs) of glioma in *Mrc1*^+/-^, *Mrc1^-/-^*, and *Mrc1*^+/+^ mice treated with D-mannose are significantly lower compared to that of *Mrc1*^+/+^ mice at 60 min (n =3 except for n = 4 for *Mrc1*^+/-^ mice). CNR = contrast-to-noise ratio.

In contrast to the reparative role of CD206^+^ macrophages in wound healing, CD206^+^ tumor-associated macrophages (TAMs) in cancer are tumorigenic and immunosuppressive 3, 37. Thus, we next tested whether Mann2-DTPA-Gd can detect these protumor TAMs in experimental glioma despite these cells serving different roles than in injury. We observed substantial contrast enhancement post-injection at 60 min (**Figure 3A**), revealing the presence of CD206^+^ TAMs in the tumor microenvironment. Interestingly, while there was a mild increased signal with a few more intense foci within the tumor, the areas with the highest contrast enhancement formed a ring around the tumor, showing that CD206^+^ TAMs predominately surround the tumor.

To further demonstrate the specificity and efficacy of Mann2-DTPA-Gd to CD206^+^ TAMs, we performed MR imaging of glioma using *Mrc1*^+/-^ mice, *Mrc1*^-/-^ mice, and *Mrc1*^+/+^ mice with competition using D-mannose. We observed significantly decreased CNR in *Mrc1*^+/-^ mice (p =0.037, n = 4) and *Mrc1*^+/+^ mice treated with D-mannose 30 mins before Mann2-DTPA-Gd administration compared to that in the control *Mrc1*^+/+^ mice at 60 min (p = 0.007, n = 3), with *Mrc1*^-/-^ mice demonstrating the lowest signal (p = 0.002, n = 3, **Figure 3A** and** 3B**). Furthermore, due to the less dense CD206^+^ TAMs surrounding the tumor, these mice demonstrated more even distributed signals throughout the tumor area instead of the high signal enhancement between the surrounding area and tumor area as observed in the control mice (**Figure 3A**). These data collectively demonstrated the specificity and efficacy of Mann2-DTPA-Gd for detecting CD206^+^ TAMs in tumors.

### MR imaging of ischemic stroke

**Figure 4:**
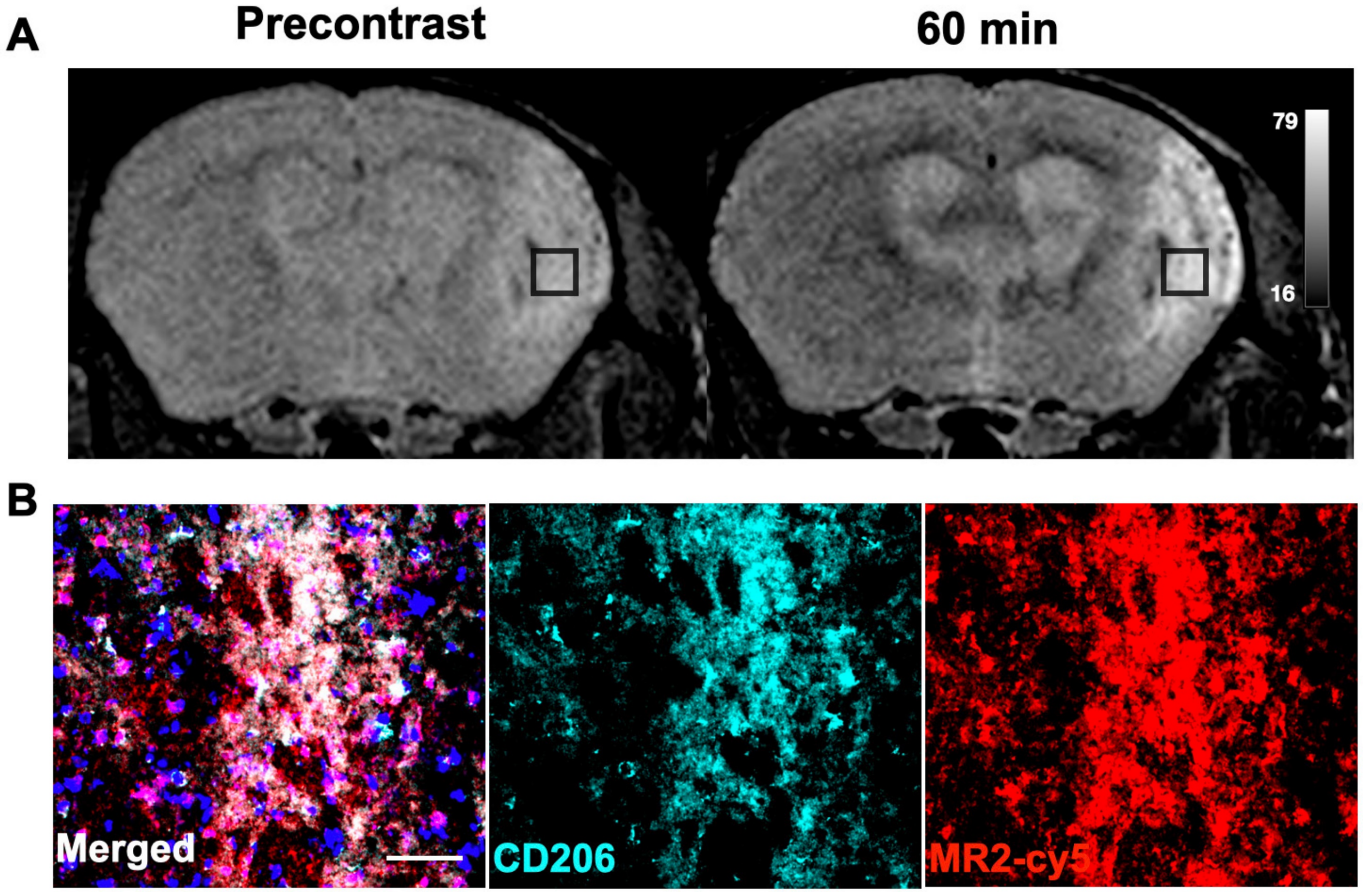
** MR imaging of ischemic stroke with Mann2-DTPA-Gd and histology. A**) MR imaging of Mann2-DTPA-Gd at 60 min was much higher than that of the pre-contrast in ischemic stroke mice on day 11. **B**) Fluorescent signal with MR2-cy5 correlated well with CD206 from immunofluorescent staining of the stroke brain sections (green, anti-CD206; red, MR2-cy5; blue, DAPI). Scale, 20 µm. CNR = contrast-to-noise ratio.

Emerging studies have shown that CD206^+^ microglia/macrophages play protective/reparative roles in neurological diseases such as stroke 7, 38, Alzheimer's 39, and multiple sclerosis 40, among others. We next examined if Mann2-DTPA-Gd MRI can detect activated microglia/macrophages in ischemic stroke at the subacute reparative stage 38. MR imaging of Mann2-DTPA-Gd on day 11 post-ischemic stroke found a marked increase in signal at 60 min compared to that of the pre-contrast images (**Figure 4A**). To further confirm the imaging results, we performed an immunofluorescence study after imaging with the fluorescent agent MR2-cy5 and found that the fluorescent signal of MR2-cy5 corroborated well with that of CD206 immunostaining in the infarcted areas of the brain (**Figure 4B**). Colocalization analysis between CD206 and MR2-cy5 using JACoP in ImageJ showed that Pearson's correlation coefficient (PCC) was 0.80, and Manders' overlap coefficients were M1 = 0.65 and M2 = 0.77, indicating strong colocalization between CD206 and MR2-cy5 (**Figure S11**) 41.

### Development of the macrocyclic-based MRI agent MannGdFish

#### Synthesis and *in vitro* validation

**Scheme 4:**
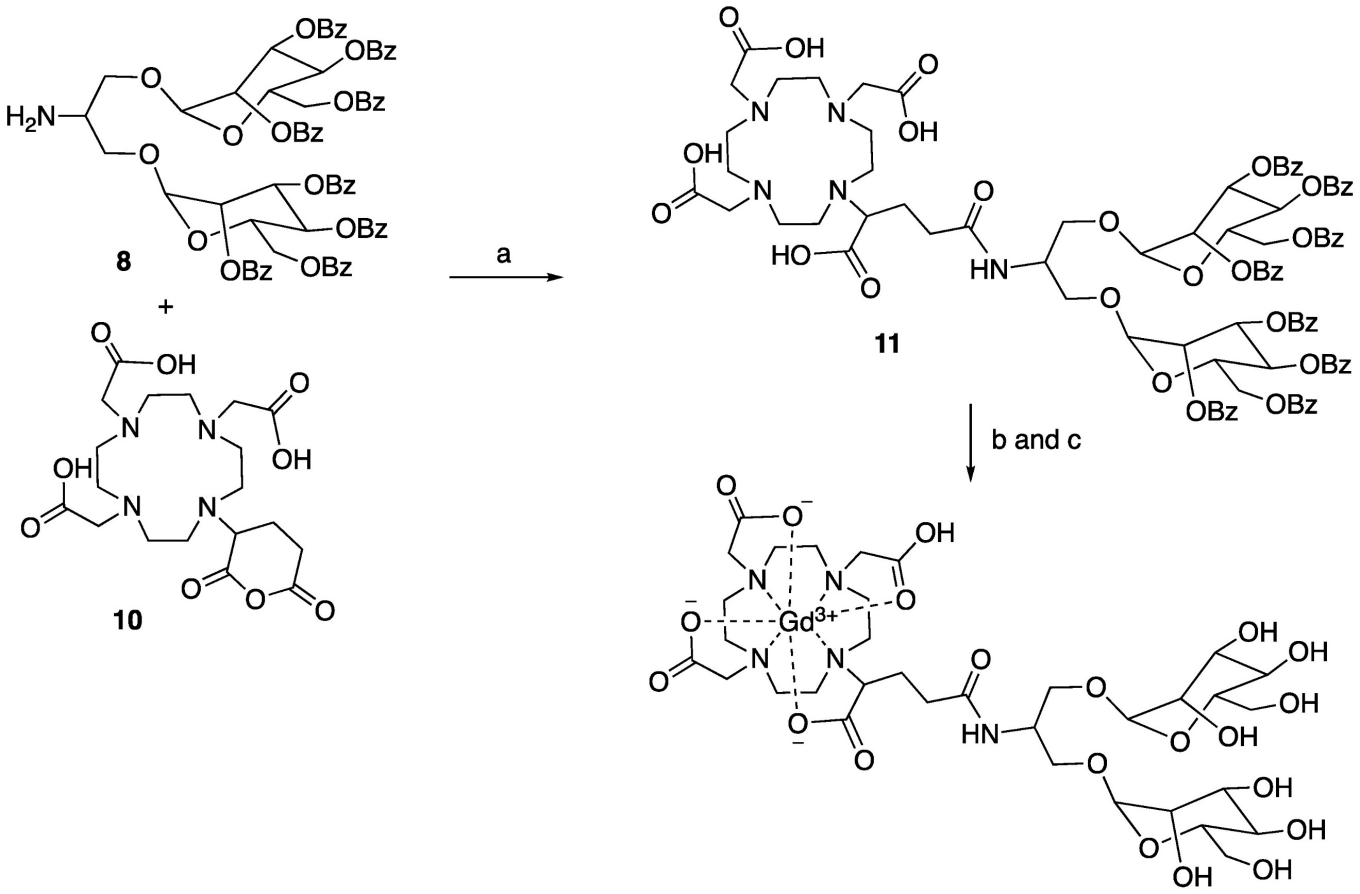
a) DIPEA, DMSO, 70 °C, overnight, 71%; b) NaOMe/MeOH, rt; c) GdCl_3_, pH 5.5, sodium acetate buffer, 45% for the last two steps.

**Figure 5:**
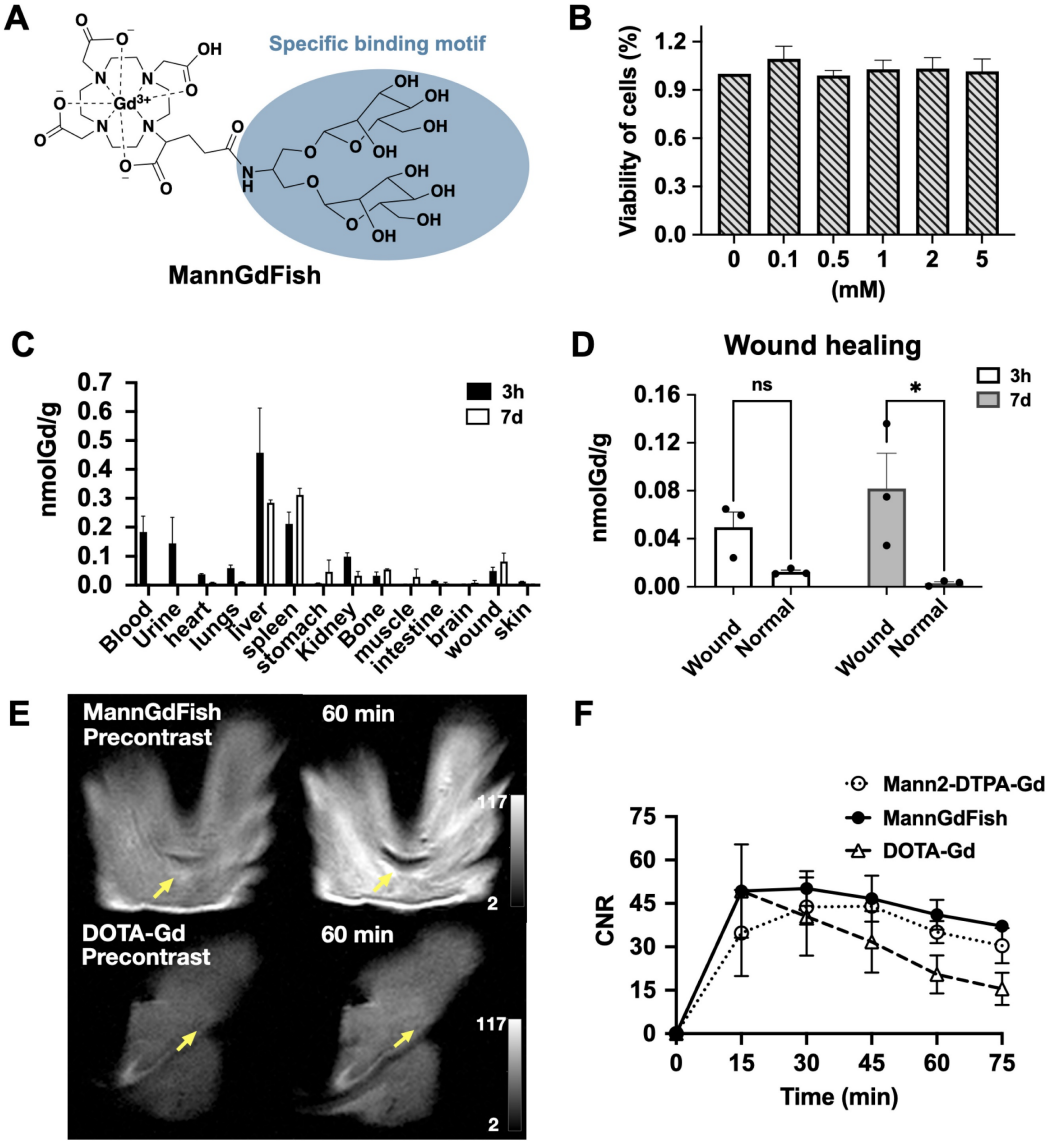
** Development of MannGdFish. A)** Structure of MannGdFish.** B)** Cytotoxicity of MannGdFish in MTT assays. No cytotoxicity was observed at a concentration as high as 5 mM (n = 3). **C)** Biodistribution of MannGdFish at 3 h and on day 7 after MannGdFish injection (24 hours after wound induction) detected by ICP-MS in a mouse model of subcutaneous wound healing. **D)** ICP-MS of wound skin compared to normal skin demonstrated much higher accumulation of MannGdFish in wound skin on day 7 (Two-way ANOVA, ns: no significant difference, p = 0.26; *, p = 0.017). **E)** Representative MR images of MannGdFish and DOTA-Gd. Signal of MannGdFish was much higher in comparison with that of DOTA-Gd in wound healing at 60 min.** F)** MR imaging of MannGdFish demonstrated slightly higher CNRs and similar dynamic profile as that of Mann2-DTPA-Gd in wildtype wound healing mice on day 7. Both MannGdFish and Mann2-DTPA-Gd showed slower washout compared to DOTA-Gd. CNR = contrast-to-noise ratio.

MR imaging of the prototype Mann2-DTPA-Gd proved it is feasible to use MRI to track CD206^+^ macrophages. However, since Mann2-DTPA-Gd contains a linear chelator, which has less Gd stability and is thus undesirable for translation, we developed a thermodynamically more stable macrocyclic-based agent (**Figure 5A**). In addition to having a more stable DOTA chelating backbone, this agent contains a clustered two-mannose moiety derived from the same intermediate **8** as MR2-cy5 (**Figure 1A**), thus possessing similar specificity for CD206^+^ macrophages. The synthesis of the agent involves intermediate **8** coupled with DOTAGA anhydride **10** under basic conditions to provide compound **11** which underwent deprotection and chelation with GdCl_3_ to give the final product. The detailed synthesis and characterization of MannGdFish are shown in Methods **Scheme 4** and **Figure S12**. We named this agent MannGdFish since its chemical structure resembles a goldfish. The relaxivity (r_1_) of MannGdFish is 5.2 mmol^-1^s^-1^ (**Figure S13**), slightly higher than that of Mann2-DTPA-Gd (3.6 mmol^-1^s^-1^). MannGdFish demonstrated no cytotoxic effect up to 5 mM (**Figure 5B**), a dose thousands of times higher than expected for first-pass concentration in the blood (μM), in MTT assays using RAW 264.7 cells.

#### Biodistribution and pharmacokinetics of MannGdFish

We next evaluated the biodistribution of MannGdFish in mice induced with a subcutaneous wound. ICP-MS showed very little accumulation of Gd or retention in the body (< 0.5 nmol Gd per gram tissue), with liver and spleen being the major organs containing gadolinium at both 3 h and on day 7, followed by blood, urine, and kidneys (**Figure 5C**). As expected, the amount of gadolinium in the injured wound (24 h post-injury) at 3 h after MannGdFish injection was low and not significantly different compared to that in the normal skin, while the gadolinium content on day 7 was higher in the wound than that in the normal skin (**Figure 5D**), again highlighting the ability of MannGdFish to track CD206^+^ macrophages during wound healing. The blood half-life of MannGdFish for the fast phase was 0.3 min and 6.1 min for the slow phase using a two-phase exponential model (**Figure S14**).

#### *In vivo* imaging of MannGdFish in wound healing

We performed MR imaging of MannGdFish in wound healing on day 7 and compared it with that of Mann2-DTPA-Gd and the non-specific agent DOTA-Gd. The signal of MannGdFish at 60 min was much higher compared to that of DOTA-Gd (**Figure 5E** and** 5F**), consistent with binding and retention of MannGdFish to CD206^+^ cells. Unlike the slow decrease of CNR in MannGdFish and Mann2-DTPA-Gd at 45 min, the CNR of DOTA-Gd decreased rapidly after peaking at 15 min post-injection (**Figure 5F**). The CNR of MannGdFish was slightly higher compared to that of Mann2-DTPA-Gd and showed a similar kinetic profile over 75 min as that of Mann2-DTPA-Gd (**Figure 5F**). These results showed that MannGdFish has similar efficacy as Mann2-DTPA-Gd while exhibiting a significantly superior safety profile due to its macrocyclic chelating backbone, confirming it as a potential translational candidate for CD206^+^ macrophage MR imaging.

## Discussion

Activated macrophages play important roles in the innate immune response, and their diversity drives both damage and repair in many diseases 3, 4, 37, 42-45. The development of technologies to target and differentiate between the different subtypes of activated macrophages is critical to better understand the functions of these cells in diseases and to develop novel therapies targeting different subtypes of macrophages. In this study, we developed fluorescent and MRI agents to detect CD206^+^ macrophages to demonstrate our targeting approach and provide non-invasive imaging tools to track and monitor CD206^+^ macrophages. We validated the specificity and efficacy of the agents both *in vitro* in cellular assays and *in vivo* in animal models of subcutaneous wound healing, glioma, and stroke.

CD206 is a well-established surface marker for anti-inflammatory/reparative macrophages 17. D-mannose is the most common ligand for CD206. However, its binding affinity is much lower than that of clustered mannoside containing two D-mannose units (IC_50_: 5.5 mM vs. 18-23 µM) or more 28. Although single D-mannose-based imaging agents have been reported 20-22, as demonstrated in **Figure 1B**, a single mannose moiety was unable to adequately differentiate pro-inflammatory macrophages from anti-inflammatory macrophages. We overcame the specificity issue by adding clustered mannose moieties to the imaging agents, given that such clustered mannose unit has a binding affinity over 100 times higher than that of D-mannose 28. Indeed, our data demonstrated that imaging agents containing the clustered two-mannose moiety derived from the intermediate **8** in MR2-cy5 and MannGdFish (**Figure 1A** and **5A**) are specific to CD206^+^ macrophages. Furthermore, even though MR2-DTPA-Gd does not contain a clustered binding motif as MannGdFish and MR2-cy5, when the chelate wraps around Gd the two mannose moieties are brought to close proximity, simulating a clustered structure.

Normal wound healing is a highly regulated process in which activated macrophages function distinctively at different stages 2. In the early inflammatory stage post-injury (1-4 days), proinflammatory cells dominate. By days 5-10 post-injury, inflammation starts to resolve, and the reparative stage begins where anti-inflammatory and reparative macrophages become the most abundant cell type, peaking around day 7 2, 46. We showed that CD206-targeted MRI can accurately report the temporal changes of these anti-inflammatory/reparative macrophages longitudinally in wound healing, corroborated by another anti-inflammatory marker (Arg1) (**Figure 2F** and** 2G**). We also observed a slightly higher CNR signal on day 1 than on day 4, which was confirmed by a flow cytometric study (**Figure 2G**) and consistent with a previous study 38. Similarly, in a mouse model of ischemic stroke where CD206^+^ microglia and macrophages play neuroprotective roles at the subacute stage, MR2-cy5 signal correlated well with CD206 immunostaining on histological images and colocalization analysis (**Figure 4**). On the other hand, CD206^+^ macrophages can also exert detrimental effects on the host. For example, TAMs are the most abundant immune cells in the tumor microenvironment. The TAMs facilitate tumor growth, immune evasion, and metastasis, and are associated with poor prognosis 9. We showed that CD206-targeted MRI can detect CD206^+^ TAMs, differentiate *Mrc1*^+/+^, *Mrc1*^+/-^, and *Mrc1*^-/-^ mice, and track treatment changes of CD206^+^ TAMs treated by D-mannose in a mouse model of glioma (**Figure 3**).

More importantly, the critical role of activated macrophages in multiple diseases has been widely recognized and therapies targeting macrophages, especially reprogramming between different subtypes, have emerged as important strategies 9. Repolarization from a pro-inflammatory phenotype to an anti-inflammatory/reparative phenotype would favor repair and recovery in diabetic wound healing 45 and in many neurological diseases 40, 47, 48. Conversely, shifting from CD206^+^ TAMs to pro-inflammatory tumoricidal macrophages is becoming an important strategy to treat cancer 3, 13, 49-51. Given the specificity and efficacy of our agents with two-mannose moieties for CD206^+^ macrophages in wound healing, glioma, and stroke, we envision that our imaging agents could provide a valuable tool to noninvasively image and track healing and disease evolution, and to monitor macrophage-targeted therapeutic effects in experimental studies and in patients. Using the specific binding motif (**Figure 5A**) as a platform, other imaging modalities such as SPECT and PET imaging could be developed (e.g., by introducing ^111^In or ^68^Ga into the chelate of MannGdFish for SPECT or PET, respectively). Furthermore, the specific binding motif we developed could be incorporated to provide highly specific targeted delivery of therapeutic drugs to enable repolarization of CD206^+^ macrophages (e.g., in cancer and fibrosis) as therapeutic or theranostic agents.

This study has some limitations. We have shown that the MRI agents containing two clustered mannose moieties are highly specific to CD206; agents with more mannose units may provide even higher binding affinity, which needs to be further studied. However, the synthesis of imaging agents with more than two mannose units is challenging, especially for MRI agents, which require at least 100 times higher amounts than fluorescent probes. As such, the synthesis and evaluation of the fluorescent and MRI agents containing more mannose moieties are underway. Although CD206 is a well-established surface marker for activated macrophages, other cell types, such as skin keratinocytes and certain (liver) endothelial cells also express CD206 52, 53. However, in the context of an inflammatory response in deep tissues or organs we are targeting, elevated CD206 expression from the activated macrophages is the major contributor to the immune microenvironment surrounding the injury/tumor 18, 54, 55. This was confirmed by the longitudinal flow cytometric study in the wound healing model where we gated on CD11b^+^, CD45^+^, CD86^-^, and Arg1^YFP+^ cells for leukocytes 56, and the CD206-targeted MRI signals were consistent with the Arg1 flow data (**Figure 2F** and **2G**).

In conclusion, using a multi-mannose approach we designed new imaging agents to target CD206^+^ macrophages that can be used in *ex vivo* and *in vivo* settings at different scales. We demonstrated the specificity and efficacy of these agents both *in vitro* and *in vivo* in animal models of subcutaneous wound healing, glioma, and stroke. We also developed the macrocyclic MRI agent MannGdFish with favorable pharmacokinetic and biodistribution properties, making it a potential translational candidate. Finally, in addition to fluorescent and MRI agents, this approach could be used as a platform to introduce other imaging modalities or therapeutic moieties for theranostic applications.

## Supplementary Material

Supplementary figures.

## Abbreviations

MI: myocardial infarction
CD206/*Mrc1*: mannose receptor
TAMs: tumor-associated macrophages
Arg1: arginase 1
ICP-MS: inductively coupled plasma mass spectrometry
HEK293: human embryonic kidney cells
CNR: contrast-to-noise ratio
YARG: YFP-labeled arginase-1mice
