# Supplementary material for: A specific and adaptable approach to track CD206+ macrophages by molecular MRI and fluorescence imaging
Source: Theranostics. 2025 Jan 1;15(3):1094–109. doi: 10.7150/thno.96488 (PMC11700851; doi:10.7150/thno.96488)
Supplement: Supplementary file 1 — Supplementary figures. [file thnov15p1094s1.pdf]

Supplemental Materials

A specific and adaptable approach to track CD206$^+$ macrophages by molecular MRI and fluorescence imaging

Cuihua Wang[#,1,2], Negin Jalali Motlagh[#,1,2], Gregory R. Wojtkiewicz[2], Hongzhi Yang[1,2], Hyung-Hwan Kim[3], John W. Chen[*,1,2]

[1] Institute for Innovation in Imaging, Department of Radiology, Massachusetts General Hospital, Charlestown, Massachusetts, 02129, MA.
[2] Center for Systems Biology, Massachusetts General Hospital and Harvard Medical School, 185 Cambridge St, Boston, MA.
[3]Stroke and Neurovascular Regulation Laboratory, Massachusetts General Hospital, Boston, MA

[#] Both authors contributed equally to this work

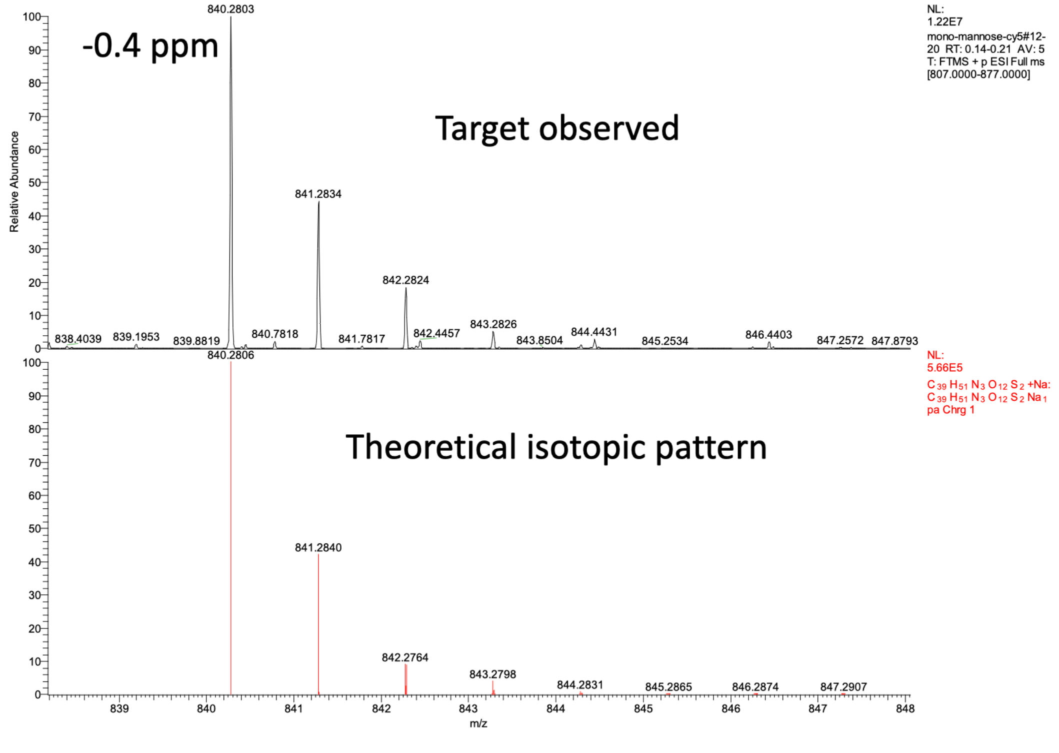

Figure S1. High resolution mass spectrometry (HR-MS) of MR1-cy5.

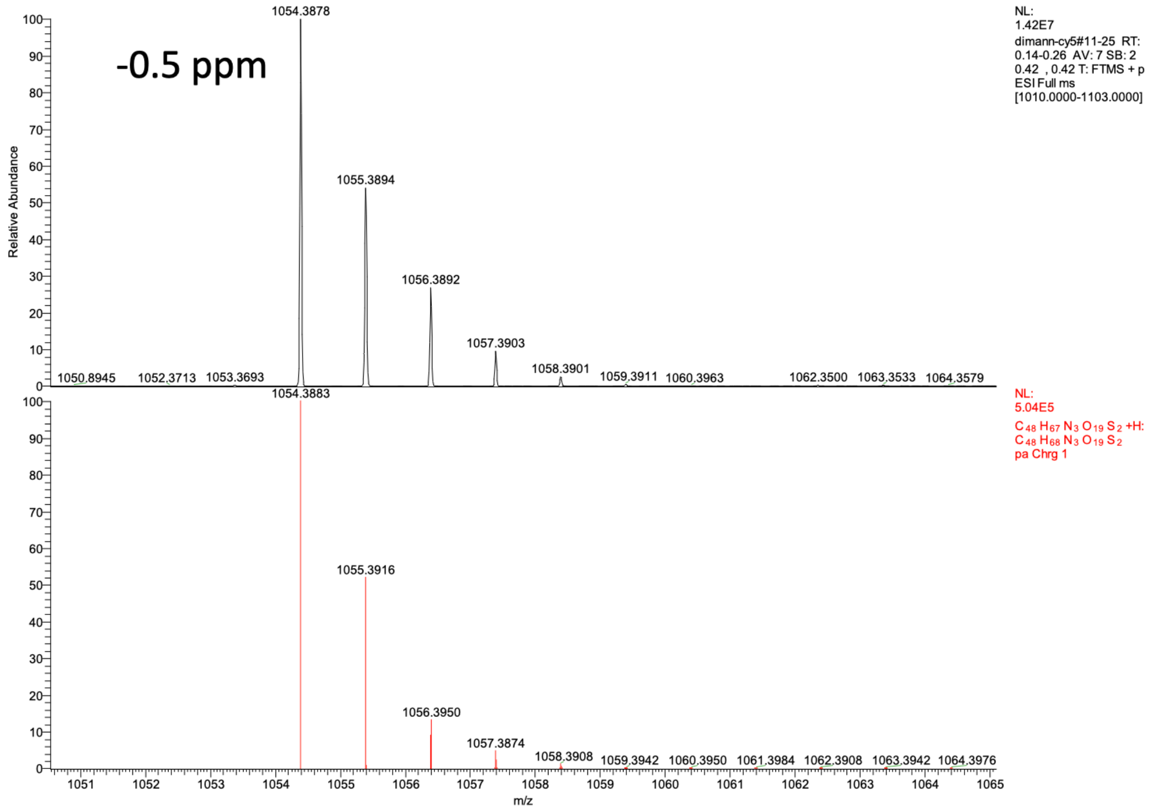

Figure S2. High resolution mass spectrometry (HR-MS) of MR2-cy5.

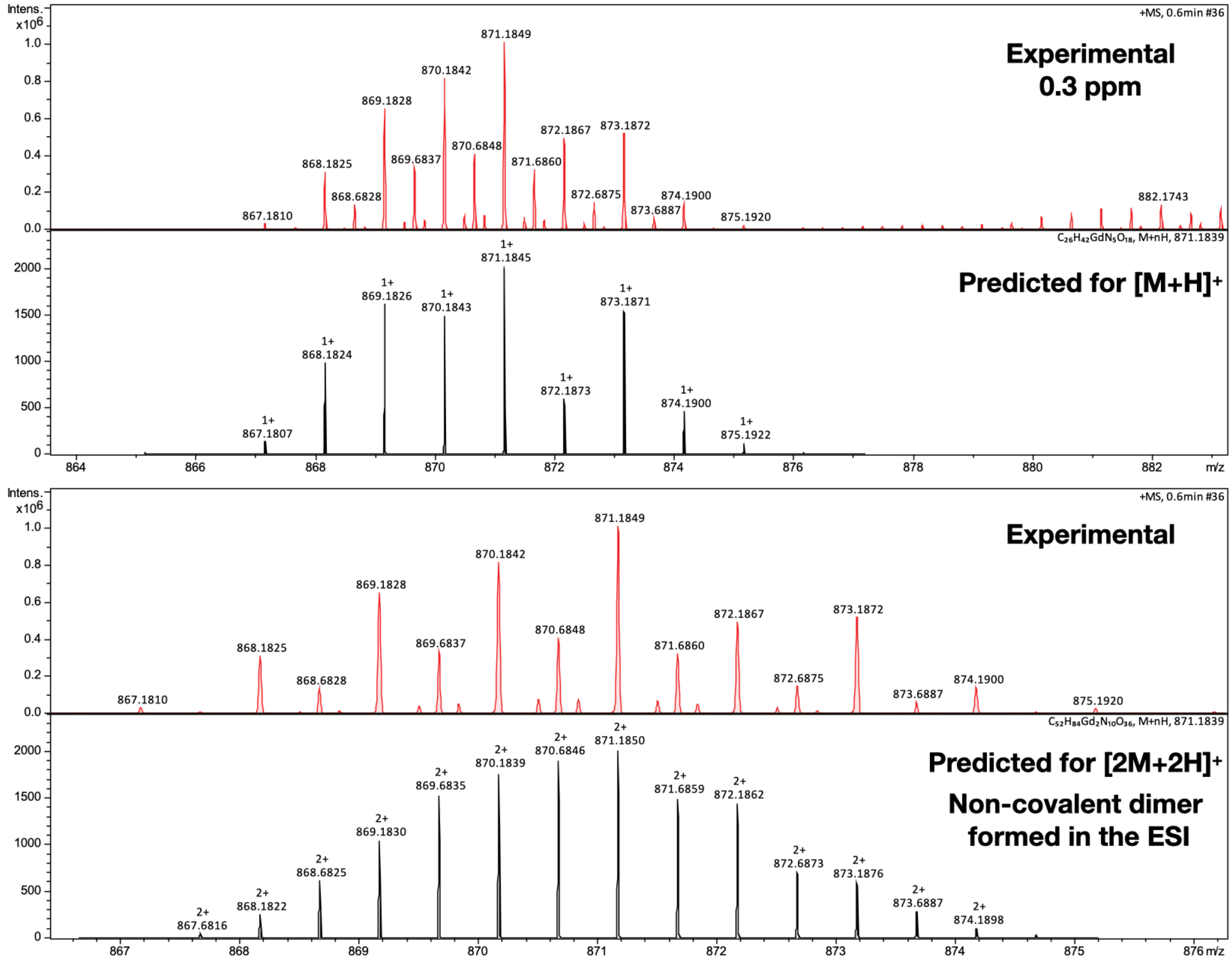

Figure S3. High resolution mass spectrometry (HR-MS) of Mann2-DTPA-Gd.

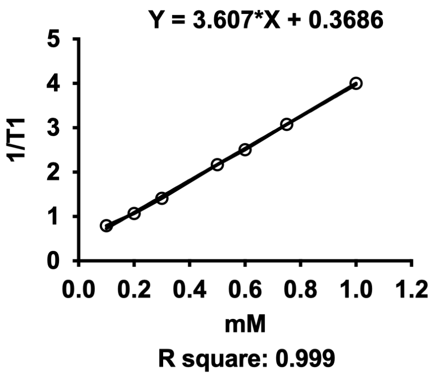

Figure S4: Relaxivity (r_1) of Mann2-DTPA-Gd.

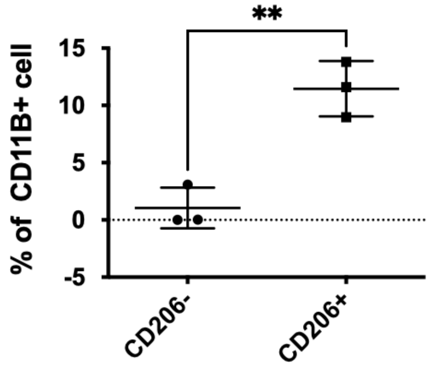

Figure S5. Validation of the differentiation of $CD206^+$ and $CD206^-$ macrophages using IFN-gamma and IL-4, respectively, from RAW 264.7 cells by flow cytometry (n = 3, unpaired t-test, **, p = 0.0038).

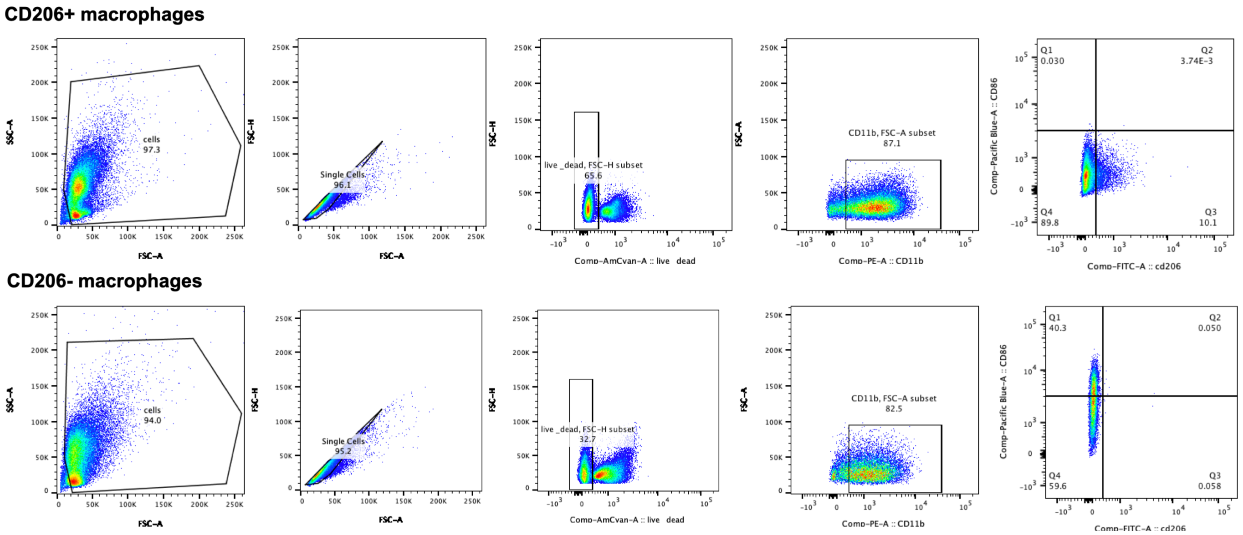

Fig. S6. Gating strategy for the differentiation RAW cells to $CD206^+$ and $CD206^-$ macrophages.

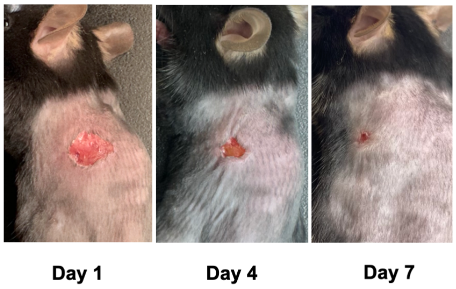

Figure S7. Mouse model of wound healing on days 1, 4, and 7.

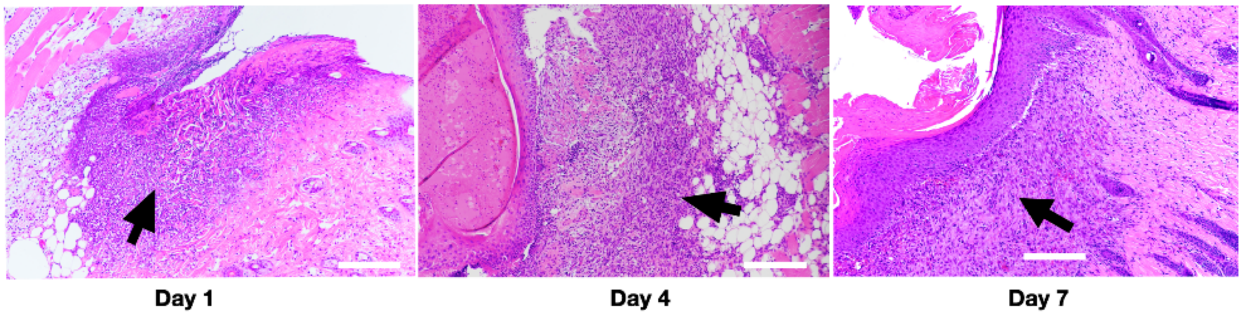

Figure S8. H&E staining of wound tissues on days 1, 4, and 7. The arrows indicate the infiltrated immune cells around the wound areas. Images: 10x, scale is 100 µm.

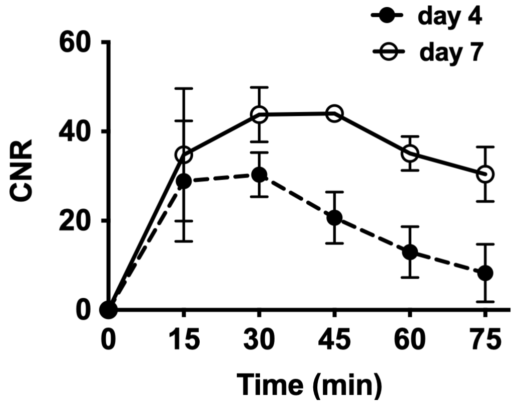

Figure S9: Contrast-to-noise ratio (CNR) of wildtype mouse at 45 min post-injection on day 7 was about 2- to 3-fold higher compared to that on day 4 after wound injury.

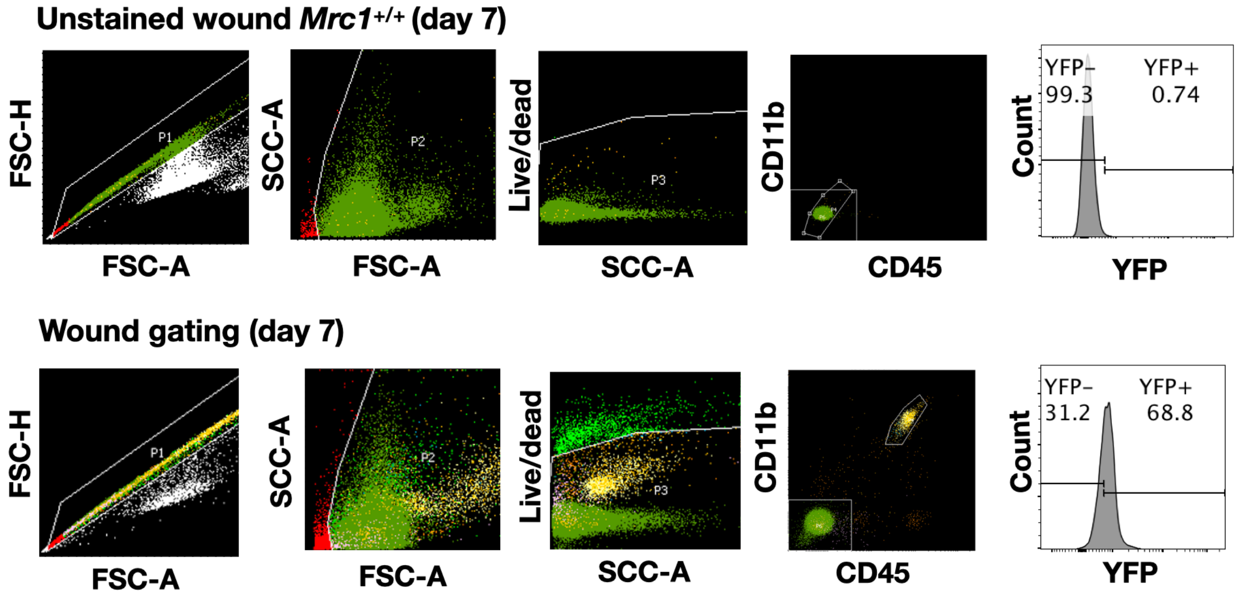

Figure S10. Gating strategy for flow cytometric analysis in the wound healing model.

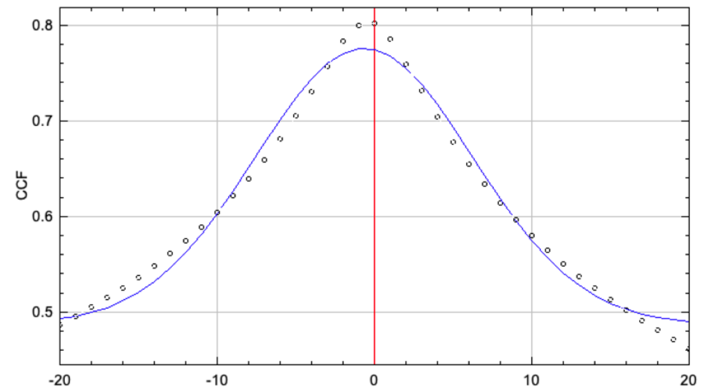

Figure S11. Van Steensel's cross-correlation function (CCF) between CD206 and MR2-cy5 in stroke.

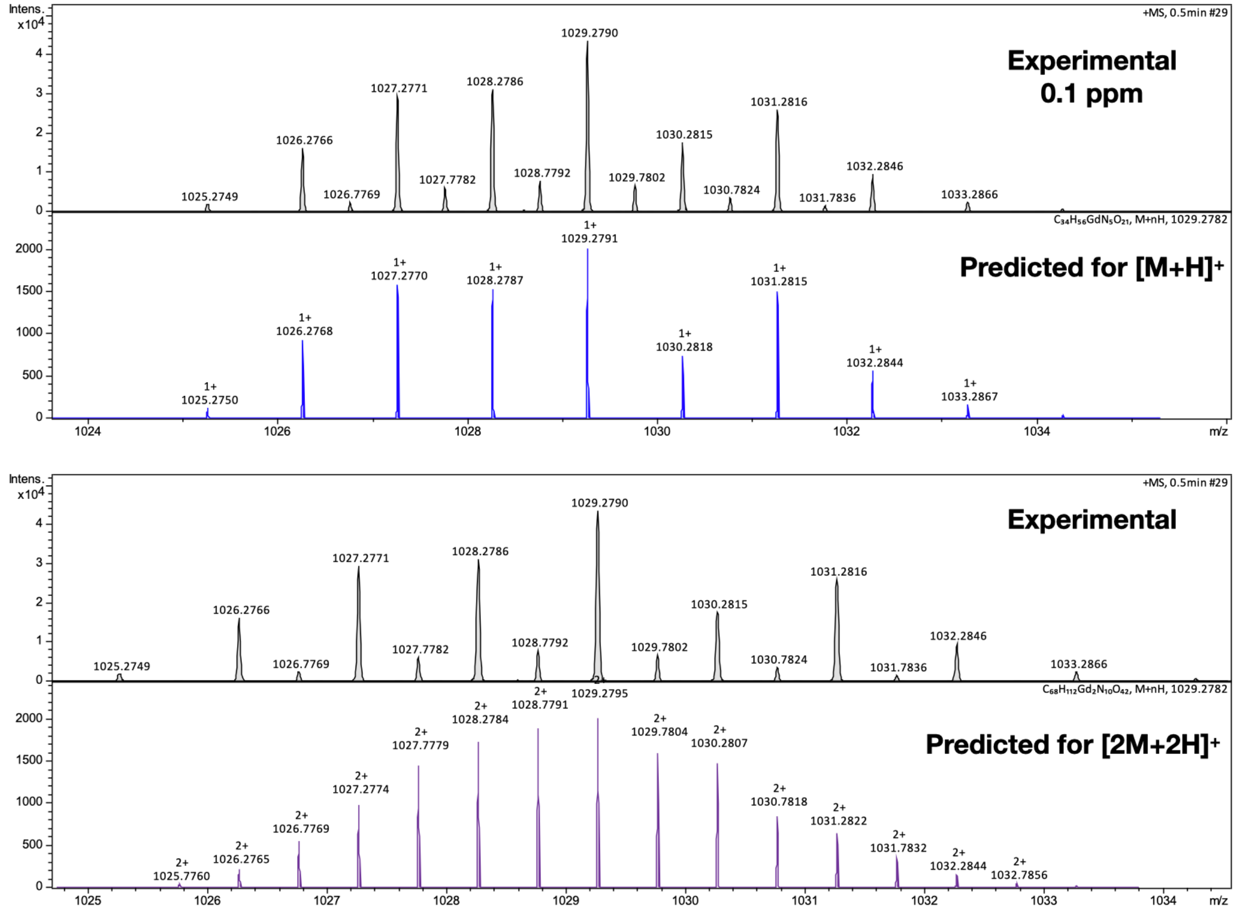

Figure S12. High resolution mass spectrometry (HR-MS) of MannGdFish.

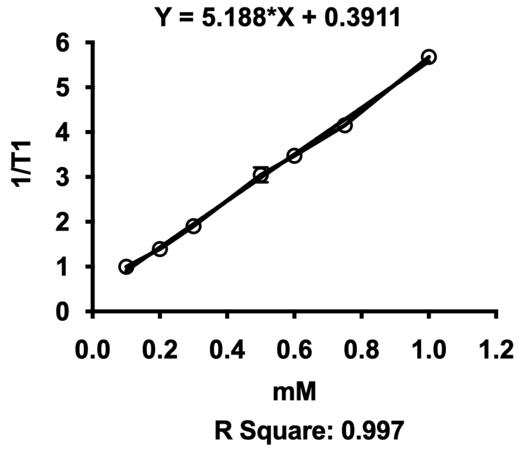

Figure S13: Relaxivity (r_1) of MannGdFish.

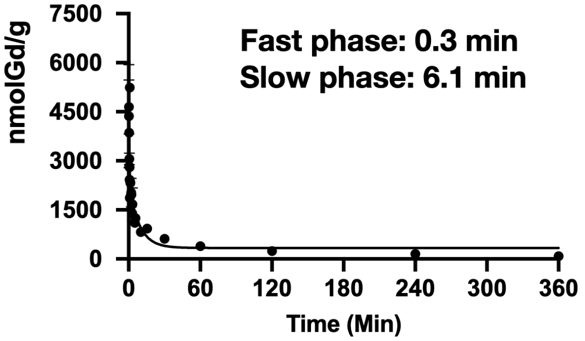

Figure S14: Blood half-life of MannGdFish detected by ICP-MS using a two-way exponential model was 0.3 min for the fast phase and 6.1 min for the slow phase.
